# Oligonucleotide Labelling Using a Fluorogenic “Click” Reaction with a Hemicarboxonium Salt

**DOI:** 10.3390/molecules181012966

**Published:** 2013-10-17

**Authors:** Marie-Pierre Maether, Kristie Lapin, Andreea Muntean, Corinne Payrastre, Jean-Marc Escudier

**Affiliations:** Modified Nucleic Acids, Laboratoire de Synthèse et Physico-Chimie de Molécules d’Intérêt Biologique (UMR 5068) Université de Toulouse (Université Paul Sabatier), Cedex 9, 31062 Toulouse, France

**Keywords:** nucleotides, modified analogues, diagnostic potentialities

## Abstract

Two fluorescent streptocyanine labelled oligonucleotides have been synthesized by a simple “click” reaction between a non-fluorescent hemicarboxonium salt and aminoalkyl functionalized thymidines within the oligonucleotide and their spectrophotometric properties have been studied.

## 1. Introduction

Oligonucleotides labelled by fluorescent dyes are widely used in numerous genomic assays from gene quantification to single nucleotide polymorphism typing [1,2,3]. The fluorescent probe can be covalently attached to the oligonucleotide either through a post-synthetic modification or by incorporation of a phosphoramidite dye during the synthesis of the oligodeoxynucleotide (ODN) [4]. The latter method suffers from a time-consuming preparation of the modified DNA building blocks that must be resistant to the acidic or basic conditions involved during the process, while the former implies the removal of the unreacted dye modifier to avoid background noise. A convenient alternative to that is the development of bioorthogonal fluorogenic reactions. This appealing methodology aims to generate fluorescent compounds from two non-fluorescent precursors that can undergo a rapid and efficient combination under mild conditions and as far as possible, with no need of any others reagents. This approach is particularly interesting because the fluorogenic reaction can be triggered through a recognition process and the “on” signal does not need the removal of a quencher from an initially fluorescent probe. Among a few possible chemical processes, the popular Cu(I) catalysed alkyne-azide cycloaddition (CuAAC) has been adapted to this purpose by developing non- or weakly-fluorescent alkynes and azides allowing the formation of highly fluorescent triazole-linked dye compounds [5]. This fluorogenic “click-on” dye synthesis has been successfully applied to nucleosides [6,7] and to ODN labelling [8,9]. The Staudinger reduction has also been used as a fluorogenic process between azido-caged coumarin-conjugated DNA and a phosphine-DNA under the guidance of a DNA template [10]. Alternatively, fluorescent cyanine dyes have been generated through a fluorogenic aldol-type reaction with or without a catalytic amine additive [11,12]. 

**Scheme 1:**
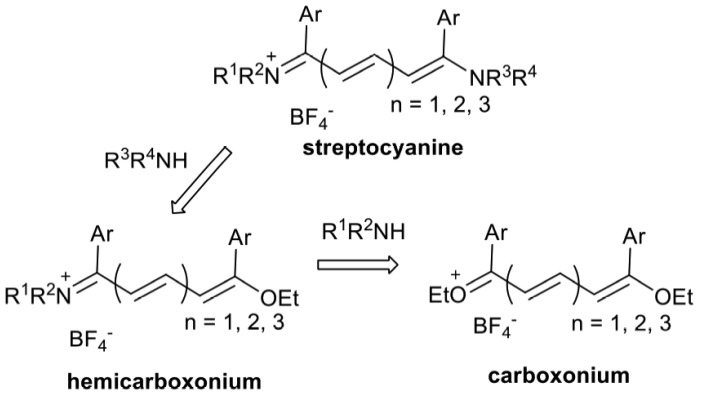
General synthetic pathway to streptocyanine dyes.

As a first step of a program aimed to develop novel fluorogenic processes aim towards labelling of biomolecules and for the *in vivo* detection of small molecules by selected nucleic acids sequences, we looked for a fluorogenic reaction that involves only two components without any additive in order to facilitate a potential *in vivo* procedure and to avoid the potential toxicity of the additive (copper for example). With these requirements in mind, we proposed a model study involving a new methodology based on the reactivity of hemicarboxonium salts towards aminoalkyl-functionalized oligonucleotides (Scheme 1 with R^3^ = ODN) that can provide access to highly fluorescent conjugates. In a buffered aqueous medium, the non-fluorescent hemicarboxonium salt precursor generates a fluorescent streptocyanine dye by a very simple “click”-type reaction through a nucleophilic substitution of the ethoxy group of the hemicarboxonium by the primary amine function of the target.

Furthermore, we evaluated the behavior of the newly synthesized streptocyanine-DNA adduct in function of the position of the dye label within the duplex, either directed towards the minor or towards the major groove of the double helix with DNA or RNA counterparts with regards to single nucleotide polymorphism (SNP) discrimination. 

## 2. Results and Discussion

The proposed post-synthetic labelling of oligonucleotides relies on the versatile methodology developed for streptocyanine dye synthesis (Scheme 1). Reaction of penta-, hepta- or nonacarbon chain carboxonium salts with various nitrogen nucleophiles (amines, imines, hydrazines, and hydrazones) lead to a key hemicarboxonium intermediate [13] that can be further combined with other amines to provide the corresponding fluorescent streptocyanines that have been fully characterized by spectroscopic techniques and X-ray diffraction analysis [14,15,16]. This allows the design of dyes in which the absorption wavelengths can be tuned across the visible and near-infrared spectrum by changing the length of the conjugated chain from three to five methine units and the nature of the terminal substituents with high extinction molar coefficients in the range of 50,000 to 250,000 mol^−1^·L·cm^−1^. The streptocyanine fluorescence properties have been used to study the diffusion of single dye molecules in the nanoporous network of sol-gel glasses [17]. Thanks to the hemicarboxonium reactivity, hybrid streptocyanines/cyclic endoperoxide or 4-aminoquinoline molecules have been prepared that exhibited good antiplasmodial activities [18].

**Scheme 2:**
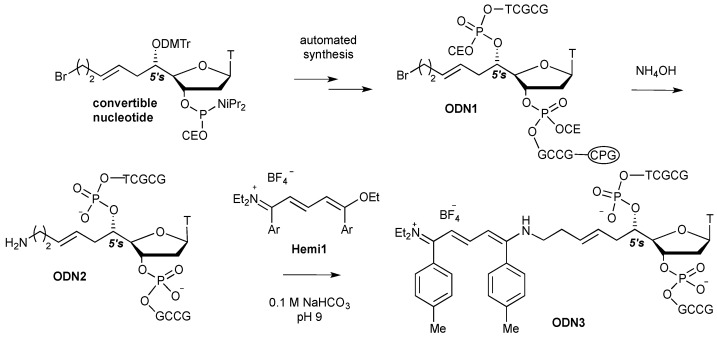
Synthetic route to streptocyanine dye labelled oligonucleotide **ODN3**. DMTr: dimetoxytrityl; CEO: cyanoethyloxy; CPG: controlled pore glass.

**Figure 1:**
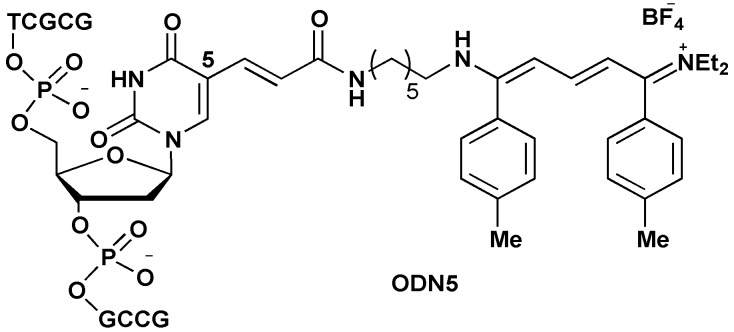
Streptocyanine dye installed on 5-position of thymidine (**ODN5**).

A model decamer sequence 5'-d(GCGCTTGCCG) was chosen because of its extinction molar coefficient of around 84,400 mol^−1^·L·cm^−1^ (λ = 260 nm) that was expected to be in the same range with the future streptocyanine one at its absorption maximum. Moreover this sequence exhibited many extracyclic amine functions that should demonstrate the chemoselectivity of the proposed reaction in favor of aliphatic amines. Two dye labelled 5'-d(GCGCTT*GCCG) oligonucleotides were prepared, **ODN3** by means of a 5'-C-convertible – **ODN1** [19] (Scheme 2) and **ODN5** (Figure 1) from **ODN4** prepared with the commercially available amino modifier uridine (amino-C6dT). Molecular modeling indicated that in the former, the pendant substitution was directed towards the minor groove [19] whereas for the latter the substitution on the 5-position of the base moiety pointed out towards the major groove when the duplex were formed with the complementary strand. 

The **ODN2** and **ODN4** derivatives were obtained by automated synthesis according to the standard phosphoramidite technology [20]. The primary alkylamine function of **ODN2** was generated by displacement of the bromine of the convertible nucleoside during the ammonia treatment for the cleavage and protective groups removal of the oligonucleotide [21,22].

The hemicarboxonium salt 1-ethoxy-5-diethylamino-1,5-bis(4-methylphenyl)-penta-1,3-dienylium tetrafluoroborate, denoted as **Hemi1**, was synthetized from the corresponding carboxonium salt by addition of one equivalent of diethylamine and was isolated in pure form after recrystallization from ethanol according to a well-established procedure [13]. 

As expected, when unprotected and unmodified nucleosides exhibiting free exocyclic amine functions were reacted with **Hemi1**, no streptocyanine dye formation was detected. Importantly, prior to undergoing the click reaction with **Hemi1**, amino substituted **ODN2** and **ODN4** had to be desalted in order to remove the ammonium counter ions and to replace them with triethylammonium ones because the former were reactive towards **Hemi1** under the reaction conditions. In 0.1 M aqueous NaHCO_3_ solution at pH 9, 20-fold excess of **Hemi1** in DMSO cleanly reacted at 25 °C with **ODN2**, whereas reaction with **ODN4** need a warm up to 40 °C to furnish the corresponding ODN/streptocyanine conjugates **ODN3** and **ODN5**, respectively. 

**Figure 2:**
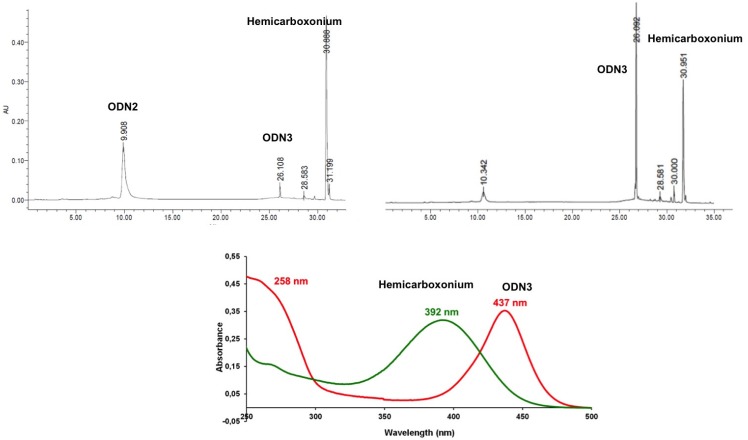
RP-HPLC monitoring of the reaction between ODN3 and Top left: RP-HPLC profile of the “click” formation of the conjugate **ODN3** at *t* = 0, Top right: *t* = 1 h (X-Bridge OST C18 2.5 μm, 4.6 × 50 mm, A: ACN; B: TEAA 50 mM, pH 7 from 5% of A to 80% in B. Bottom: UV spectra of **Hemi1** (green) and of **ODN3** (red) in pH 7 phosphate buffer.

The reaction was easy to monitor by reverse phase RP-HPLC with UV-Vis detection (Figure 2). First because the streptocyanine induced a lipophilic character to the conjugate (t_r_ = 26.1 min) in comparison with the starting ODN (t_r_ = 9.9 min) and second because the streptocyanine UV absorption (λ_max_ = 437 nm) takes place at about a 45 nm red shift in comparison to **Hemi1** (λ_max_ = 392 nm, t_r_ = 30.9 min). Therefore, the conjugates **ODN3** and **ODN5** were characterized by an extra band at 437 nm in addition to the usual nucleic acid band at 260 nm. It was clear that the reaction ran to completion for the synthesis of **ODN3** and **ODN5** in a quantitative yield since no other peaks with the UV characteristics (at λ 260 nm) of a nucleic acid were detected. Moreover the UV spectra of the conjugates showed two bands at 260 and 437 nm that gave indications on the molar extinction coefficient of the streptocyanine moieties. The calculated molar extinction coefficients of **ODN3** and **ODN5** at 260 nm are 84,400 mol^−1^·L·cm^−1^ and the band corresponding to the dyes at 437 nm were 24% lower in intensity, that led us to estimate the corresponding extinction molar coefficient values around 64,000 mol^−1^·L·cm^−^^1^ which were in the range of molar extinction coefficient values measured for streptocyanine dyes previously synthesized from aliphatic amines [13].

**Figure 3:**
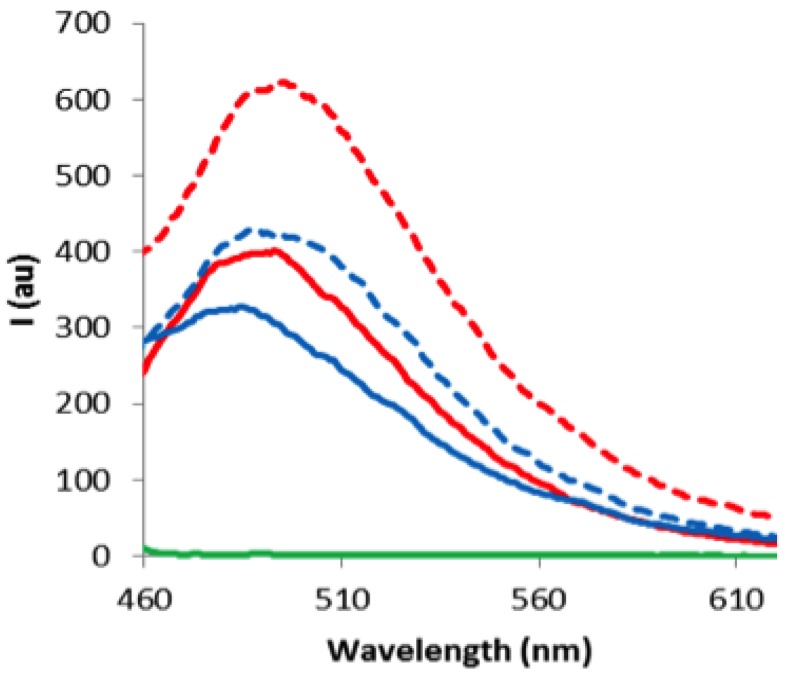
Fluorescence emission spectra (λ_ex_ = 420 nm), c = 1.49 10^−6^ M (10 mM Na_2_HPO_4_, 100 mM NaCl, 1 mM EDTA, pH = 7). Green: **Hemi1**; red: ss **ODN3**; doted red: ds **ODN3**; blue: ss **ODN5** and doted blue: ds **ODN5**.

The reaction work-up consisted in a liquid/liquid extraction of each component. The aqueous reaction medium was washed with methylene chloride to remove the excess of **Hemi1** while dye-labelled ODNs remain in the water layer. Removal of the solvent and desalting gave the labelled **ODN3** or **ODN5** that were characterized by MALDI-TOF and UV-Vis techniques. Mass spectrometry analysis showed that only one streptocyanine was formed, which confirmed that the amine functions of the nucleic bases were unreactive towards **Hemi1** (see SI). UV-Vis and fluorescence studies of these conjugates in single (ss) and double strand (ds) contexts were carried out in pH 7 phosphate buffer (10 mM Na_2_HPO_4_, 100 mM NaCl, 1 mM EDTA, see Supplementary Information). The dye conjugation induced a weak destabilizing effect of −1 °C in duplexes formed between **ODN3** or **ODN5** with complementary DNA strand 5'-d(CGGCAAGCGC) whereas with complementary RNA strand 5'-r(CGGCAAGCGC) the duplexes were destabilized by −5.2 with **ODN3** and slightly favored by +0.8 °C with **ODN5**. It is noteworthy that no bleaching was observed during the denaturation studies. Moreover, fluorescence spectra clearly showed that the conjugates exhibited nice emission bands around 500 nm, as expected for this kind of streptocyanine, whereas the starting **Hemi1** was not fluorescent (Figure 3).

Only slight differences were observed in the absorption and fluorescence emission wavelengths of the streptocyanine moiety between **ODN3** and **ODN5** either in single strand (λ_abs_ = 437.0 and 435.5 nm; λ_em_ = 490.0 and 485.0 nm, respectively) or in a double stranded context (λ_abs_ = 438.5 and 435.5 nm; λ_em_ = 500.0 and 500.0 nm, respectively, Figure 3). On the other hand, fluorescence intensity was 1.5 fold-enhanced from single to double strand in both cases and the fluorescence of **ODN3** was always higher than that of **ODN5** which suggested the relative impact of the position of the dye on the chain in favor of a 5'-C-positioning. 

The streptocyanine-oligonucleotides **ODN3** and **ODN5** have been evaluated for their ability to discriminate single-nucleotide mismatches in DNA/DNA or DNA/RNA duplexes by mean of fluorescence. DNA and RNA complementary sequences 5'-(CGGCAAT(U)CGC), 5'-(CGGCAT(U)GCGC), 5'-(CGGCT(U)AGCGC) and 5'-(CGGT(U)AAGCGC) have been designed to exhibit G/T(U), T/T(U) or C/T(U) mismatched base pairs when hybridized with **ODN3** and **ODN5**. Therefore the non-pairing nucleotide was positioned one base pair 3'-downstrean, or opposite, or one and two base pairs 5'-upstream of the dye-functionalized thymidine, respectively. Thermal denaturation data obtained for **ODN3** and **ODN5** hybridized with DNA or RNA mismatched counterparts were consistent with no disruption of the mismatch discrimination capability of the conjugates (see Supplementary Materials).

**Figure 4:**
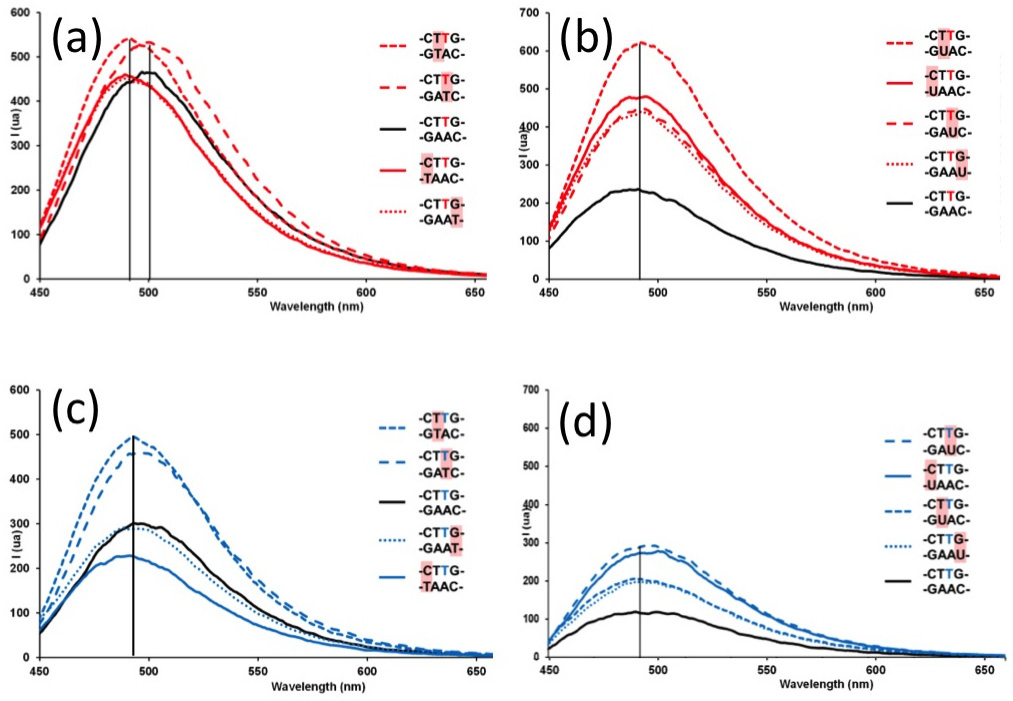
Fluorescence emission spectra (λ_ex_ = 420 nm, c = 1.49 10^−6^ M, 10 mM Na_2_HPO_4_, 100 mM NaCl, 1 mM EDTA, pH = 7) of **ODN3** (dye labelled thymidine in red) with DNA (**a**) or RNA (**b**) and of **ODN5** (dye labelled thymidine in blue) with DNA (**c**) or RNA (**d**) single mismatched counterparts, respectively.

It is noteworthy that by means of UV, fluorescence and circular dichroism spectroscopies similar free streptocyanine dyes did not exhibit any interaction with DNA or RNA, either in single stranded form nor in duplex structure. When the dye was located into the minor groove of the duplex formed with **ODN3** and DNA counterparts, only a small (around 10 nm) blue-shift of the fluorescence emission maximum was observed for the down- or upstream-mismatched base pair without significant change in fluorescence intensity when compared with the full complementary sequences (Figure 4a). With RNA-type complementary strands, there was no change in the maximum of the fluorescence emission band at 490 nm between matched and mismatched duplexes (Figure 4b). However, the fluorescence intensity was increased by two to three folds for the four mismatched sequences with respect of the fluorescence level of the fully matched duplex. The highest level of discrimination was achieved for the base mismatch immediately 5' upstream of the labelled thymidine (CTT*G/G*U*AC) whereas similar behaviors were observed for the others positioned unpaired bases (CTT*G/GAA*U*; CTT*G/GA*U*C and CTT*G/*U*AAC).

There was no significant shift observed for the position around 490 nm of the maximum of the streptocyanine fluorescence intensity when the dye was oriented towards the major groove within duplexes formed with **ODN5** and mismatched DNA or RNA counterparts (Figures 4(c,d)). A slight 1.5-fold increase of the fluorescence intensity was detected for two DNA mismatched duplex CTT*G/GA*T*C and CTT*G/G*T*AC whereas no change or even a decrease of intensity were observed for the two others cases, CTT*G/GAA*T* and CTT*G/*T*AAC, respectively. With RNA mismatch counterparts, whereas the level of fluorescence was lower than the one measured for the dye directed towards the minor groove (cf. Figure 4b and d)), a three-fold improvement of the fluorescence intensity relatively to the full complementary DNA/RNA duplex, was observed for the mismatched CTT*G/GA*U*C and CTT*G/*U*AAC and two-fold for CTT*G/GAA*U* and CTT*G/G*U*AC.

Therefore, if single mismatched base pairs were not significantly detected in DNA duplexes, streptocyanine dye-labelled ODNs showed interesting discrimination towards RNA mismatched counterparts with a more intense effect when the dye was directed towards the minor groove of the duplex. However, changes in fluorescence intensity were still very modest and insufficient for direct SNP detection when compared with DNA probes labeled with fluorene [23] or phenoxazinium [24], pyrene- or cyanine-functionalized ODNs [25,26], cyanine dye installed as base surrogates within PNA [27] or with coumarin derivatives-based detection by DNA-template fluorogenic ligation [8]. Nevertheless, these experiments underscored that the relative position of the dye within the duplex could tune the relative fluorescence intensities. Moreover, it is the first step in this direction and investigations with streptocyanines with longer polymethine chains (Scheme 1, *n* = 2 or 3) exhibiting very high extinction molar coefficients that should improve the fluorescence intensity are under process.

## 3. Experimental

### 3.1. General

Reagents and solvents were purchased from commercial suppliers (Acros, Geel, Belgium; Sigma-Aldrich, Steinheim, Germany; Chemgenes, Wilmington, MA, USA; Biosolve Chimie, Dieuze, France) and used as provided, unless indicated otherwise. 

Mass spectrometry data were recorded on a MALDI-TOF Waters Micro MX with use of THAP (2,4,6-trihydroxyacetophenone) as matrix. Analytical reverse phase high performance liquid chromatography analyses were performed on a Waters 600 coupled with a Waters 996 Photodiode array detector and a Waters 715+ auto sampler. UV experiments were recorded on a Varian CARY-300 Bio equipped with a Peltier controller and spectra were treated with the CARY Win software. Fluorescence data were recorded on a Varian CARY-Eclipse equipped with a Peltier controller and spectra were treated with the CARY–Eclipse software Needs sources of reagents, analytical instruments used, *etc*.

### 3.2. Protocol for Streptocyanine Dye Formation with Amino ODN

To a solution of ODN (0. 1 μM) in an aqueous solution of NaHCO_3_ (0.5 mL, 100 mM, pH 9) in a 1.5 mL screw neck vial was added an aliquot of a stock solution of **Hemi1** (142 μL, 20 eq) in DMSO (25.6 mg in 5 mL, 14 mM) and the mixture was placed in a dry bath (25 or 40 °C). The reaction was followed by means of HPLC with UV detection. After completion the reaction mixture was washed with dichloromethane (1 mL) five times. The supernatant was collected and salts were removed by means of filtration through a reverse phase silica gel cartridge (0.2 g, Chromafix C18ec, Macherey-Nagel, Düren, Germany) eluted with 80% acetonitrile in water. The conjugates were recovered after evaporation with a speed-vac apparatus and controlled by analytical reverse phase HPLC (Column: X-Bridge OST C18, Waters, USA, 2.5 μm, 4.6 × 50 mm, Mobile phase: A: TEEA 50 mM, pH 7 B: CH_3_CN, Gradient starting with 5% of B in A at *t* = 0 to 10% of B in A at *t* = 10 min, then 80% of B in A at *t* =15 min and for five additional min. 

## 4. Conclusions

In conclusion, streptocyanine dye formation by *in situ* reaction of a hemicarboxonium salt with a pendant aminoalkyl function installed on an oligonucleotide extends the reaction repertoire for oligonucleotide labelling. The significance of our approach is outlined by the possible orientation choice of the dye either towards the major or the minor groove of the nucleic acid duplex. This versatile “click”-type methodology could be applied in many contexts to generate fluorescent-labeled molecules. Small molecules [28] or DNA template-directed fluorogenic oligonucleotide ligation studies involving streptocyanine dye formations from non-fluorescent DNA-hemicarboxonium conjugates are underway [29,30]. 

## Supplementary Materials

A supplementary file with HPLC profiles of the formation of **ODN5**, UV spectra of **ODN3** and **ODN5** in single or double strand, mass spectra of **ODN2, ODN3**, **ODN4 and ODN5** and thermal denaturation data of **ODN3 and ODN5** within a duplex, is provided. They can be accessed at: http://www.mdpi.com/1420-3049/18/10/12966/s1.
