# Supplementary material for: Oligonucleotide Labelling Using a Fluorogenic “Click” Reaction with a Hemicarboxonium Salt
Source: Molecules. 2013 Oct 17;18(10):12966–76. doi: 10.3390/molecules181012966 (PMC6270631; doi:10.3390/molecules181012966)
Supplement: Supplementary file 1 [file molecules-18-12966-s001.pdf]

Supplementary Materials

Contents

S1: HPLC profile of **ODN3** and **ODN5** formation

S2: UV/vis and emission fluorescence spectra of **ODN3** and **ODN5** in single or double strand

S3: Mass spectra of **ODN2**, **ODN4**, **ODN3** and **ODN5**

S4: Thermal denaturation data of **ODN3** and **ODN5** within duplex

S1: HPLC Profile of ODN3 and ODN5 Formation.

HPLC Conditions:

Column: X-Bridge OST C18 2.5µm, 4.6x50 mm

Mobile phase: A:TEEA 50 mM, pH 7 B: CH_3CN

Gradient:

t (min)	A(TEEA)	B(CH_3CN)
0	95	5
10	90	10
15	20	80
20	20	80
22	95	5

ODN3 t = 0 h, 25 °C, (detector λ = 260 nm)

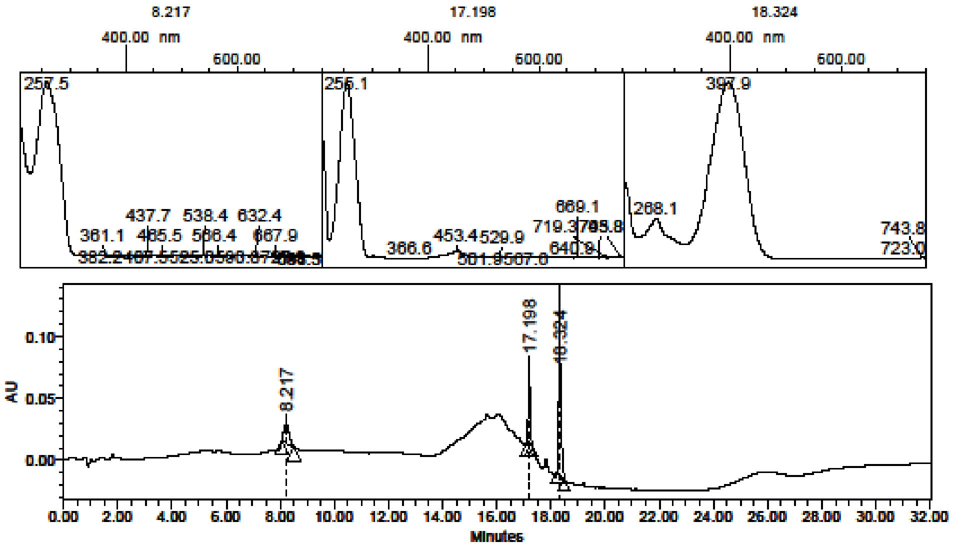

260nm

Pureté et comparaison avec la lirairie

	Name	RT	Area	% Area	Purity1 Angle	Purity1 Threshold
1		8.217	191385	15.81		
2		17.198	293519	24.25		
3		18.324	725268	59.93		

ODN3 t = 0 h, 25 °C, (detectorλ = 390 nm)

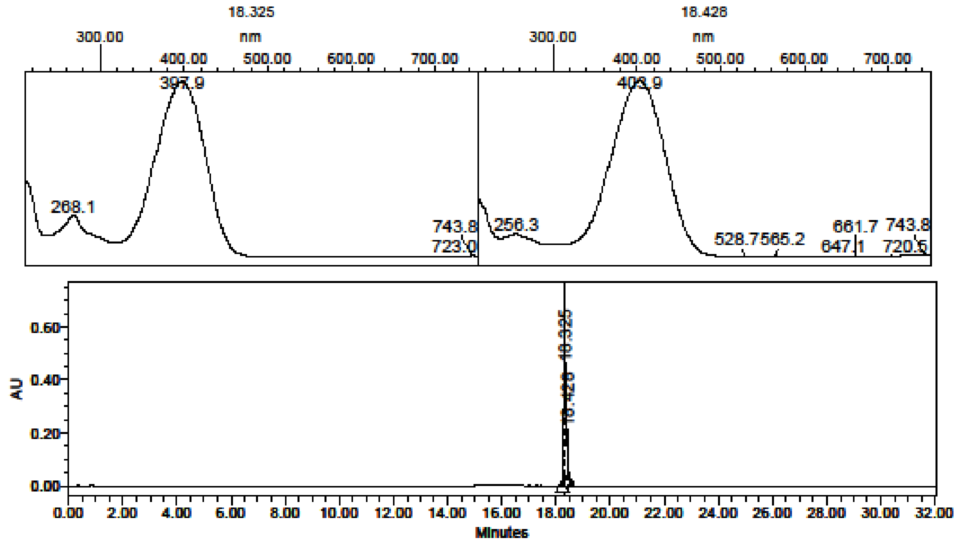

390nm

Pureté et comparaison avec la lirairie

	Name	RT	Area	% Area	Purity1 Angle	Purity1 Threshold
1		18.325	3369582	93.98		
2		18.428	215978	6.02		

ODN3 t = 0 h, 25 °C, (detectorλ = 440 nm)

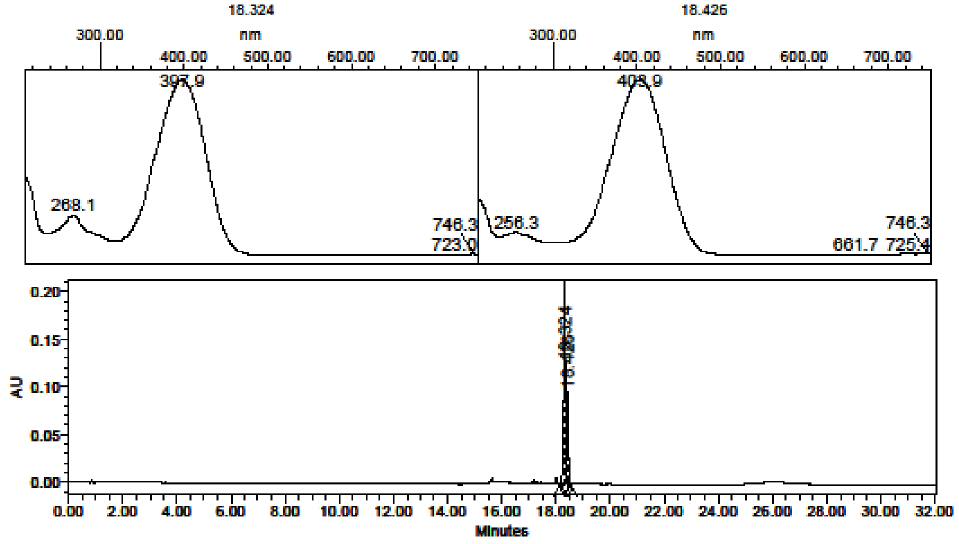

440nm

Pureté et comparaison avec la lirairie

	Name	RT	Area	% Area	Purity1 Angle	Purity1 Threshold
1		18.324	1007584	74.28		
2		18.426	348845	25.72		

ODN3 t = 1 h, 25 °C, (detectorλ = 260 nm)

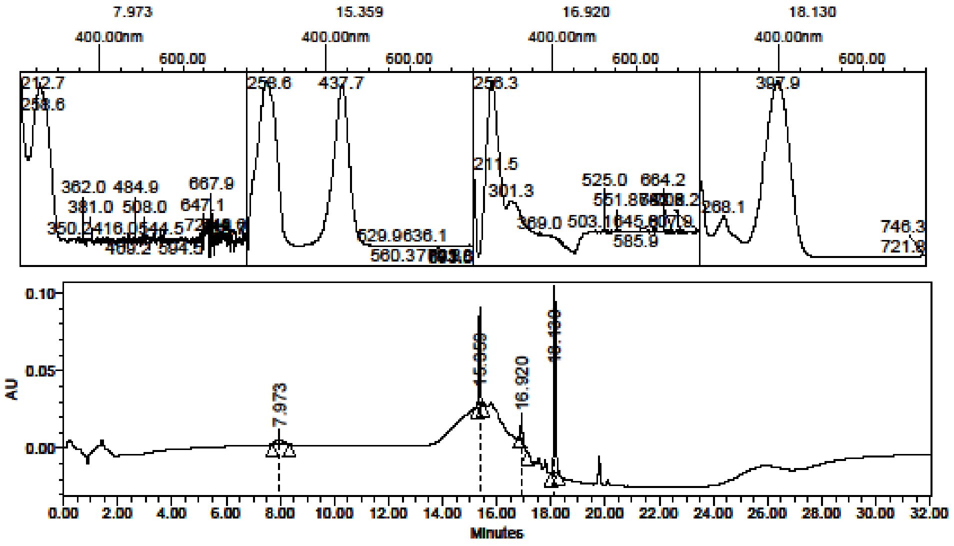

260nm

Pureté et comparaison avec la lirairie

	Name	RT	Area	% Area	Purity1 Angle	Purity1 Threshold
1		7.973	51269	5.87		
2		15.359	216215	24.77		
3		16.920	54280	6.22		
4		18.130	551261	63.14		

ODN3 t = 1 h, 25 °C, (detectorλ = 390 nm)

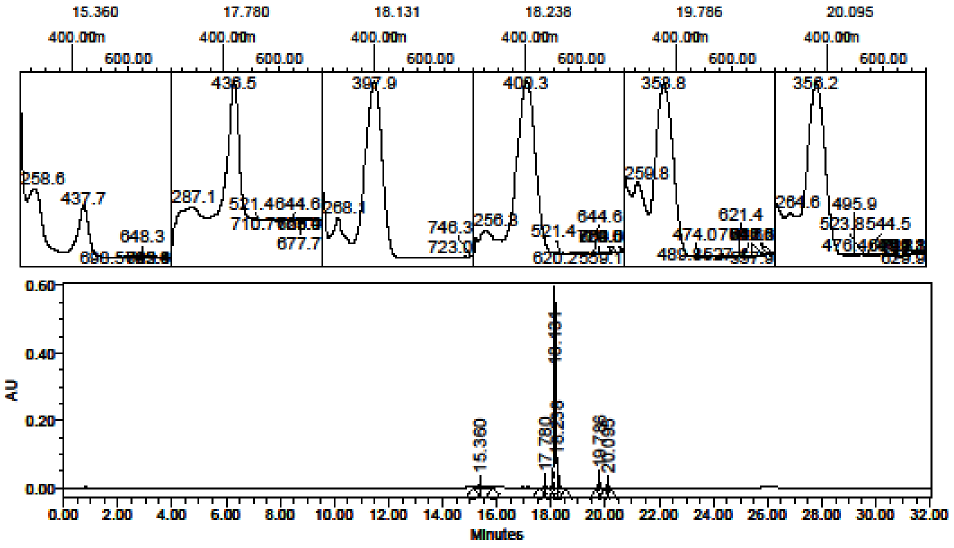

390nm

Pureté et comparaison avec la lirairie

	Name	RT	Area	% Area	Purity1 Angle	Purity1 Threshold
1		15.360	154199	4.64		
2		17.780	48580	1.46		
3		18.131	2726216	82.06		
4		18.238	246110	7.41		
5		19.786	104656	3.15		
6		20.095	42283	1.27		

ODN3 t = 1 h, 25 °C, (detectorλ = 440 nm)

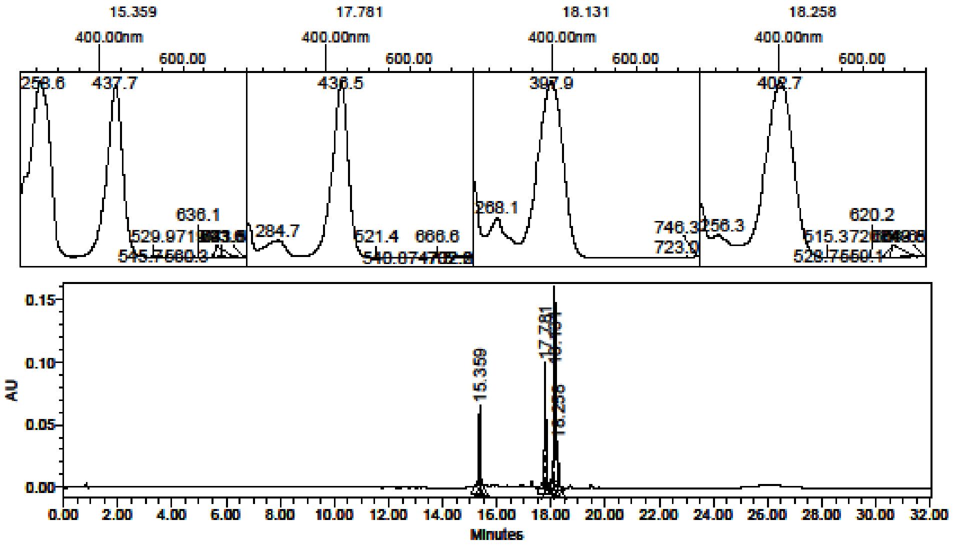

Pureté et comparaison avec la lirairie

	Name	RT	Area	% Area	Purity1 Angle	Purity1 Threshold
1		15.359	198531	14.78		
2		17.781	360985	26.88		
3		18.131	736310	54.83		
4		18.258	47145	3.51		

purified ODN3 purified (detector λ = 260 nm)

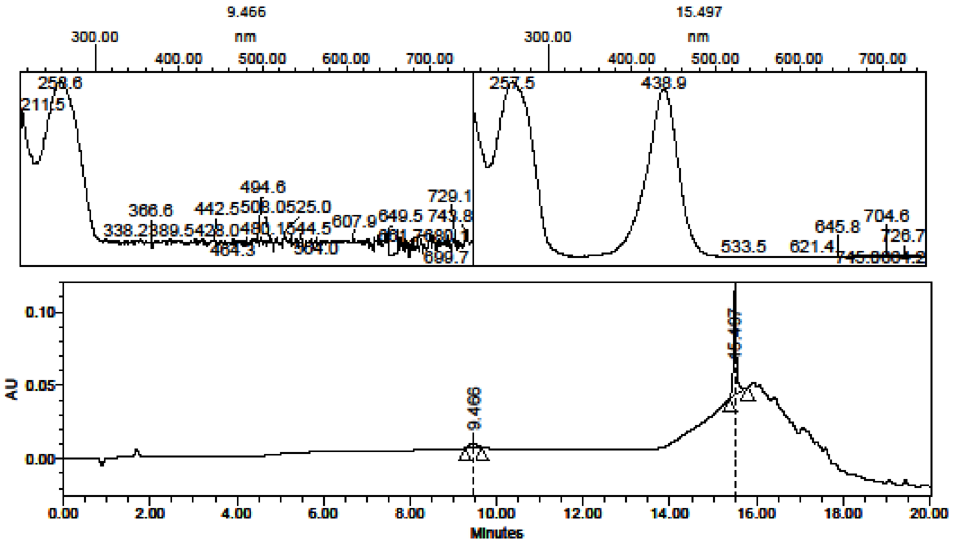

Pureté et comparaison avec la lirairie

	Name	RT	Area	% Area	Purity1 Angle	Purity1 Threshold
1		9.466	35413	9.56		
2		15.497	334981	90.44		

purified ODN3 purified (detector λ = 390 nm)

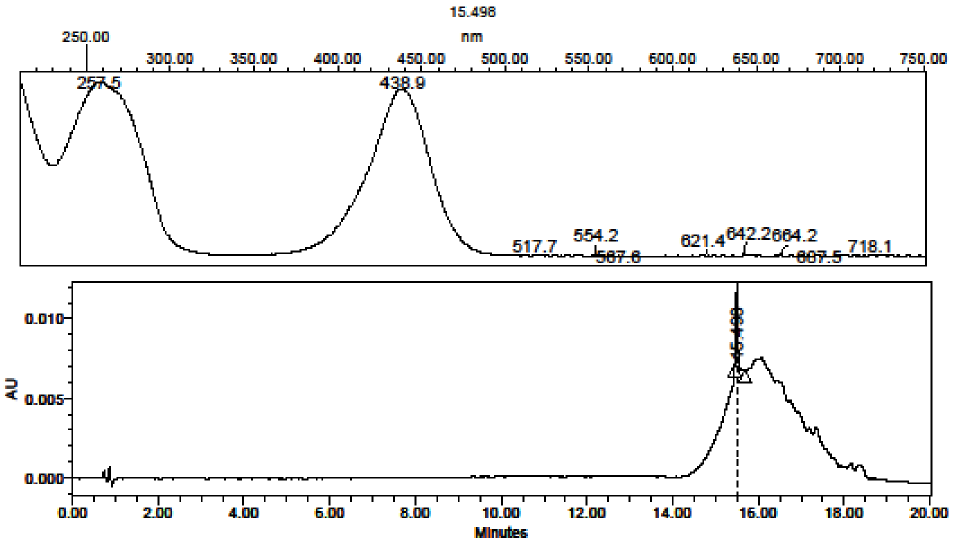

Pureté et comparaison avec la lirairie

	Name	RT	Area	% Area	Purity1 Angle	Purity1 Threshold
1		15.498	15920	100.00		

purified ODN3 purified (detector λ = 440 nm)

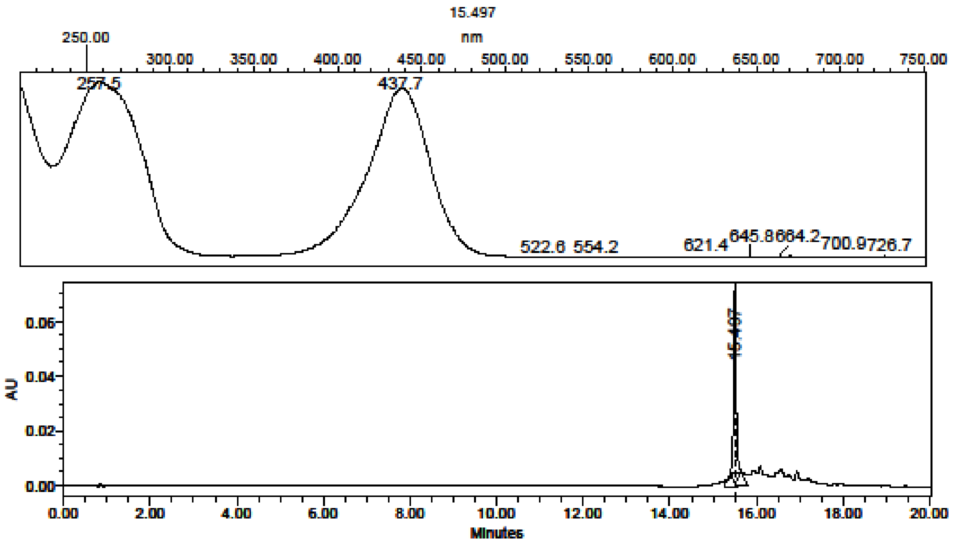

Pureté et comparaison avec la lirairie

	Name	RT	Area	% Area	Purity1 Angle	Purity1 Threshold
1		15.497	281115	100.00		

ODN5 t = 0 h, 25 °C, (detector λ = 260 nm)

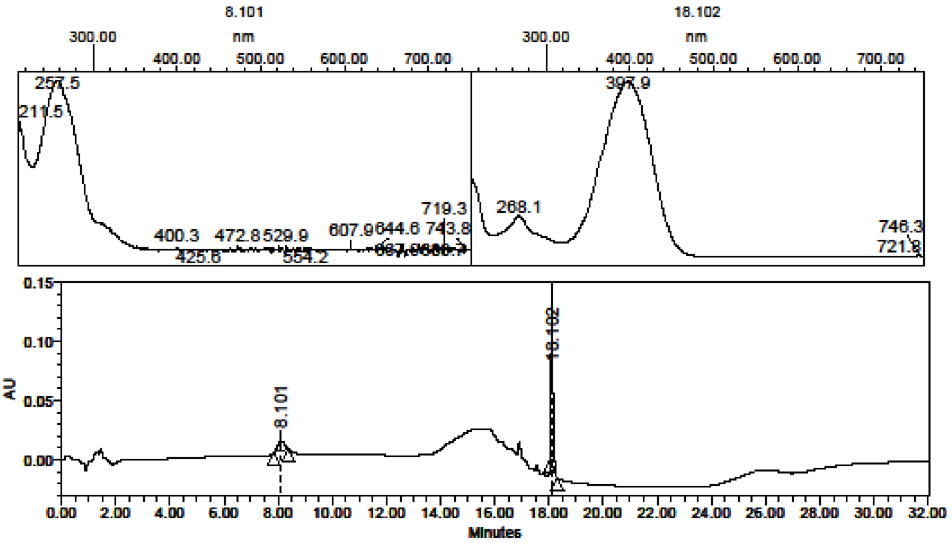

260nm

Pureté et comparaison avec la lirairie

	Name	RT	Area	% Area	Purity1 Angle	Purity1 Threshold
1		8.101	142813	17.39		
2		18.102	678501	82.61		

ODN5 t = 0 h, 25 °C, (detector λ = 390 nm)

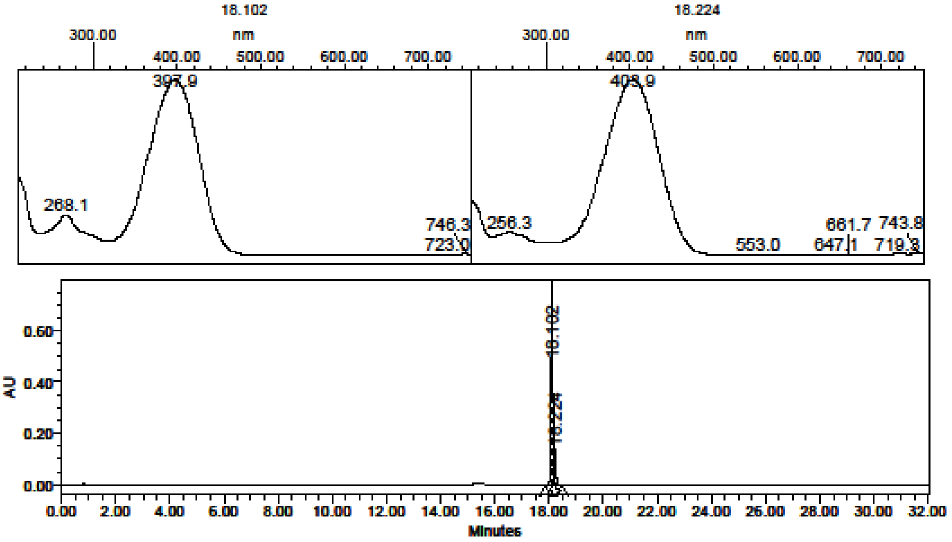

390nm

Pureté et comparaison avec la lirairie

	Name	RT	Area	% Area	Purity1 Angle	Purity1 Threshold
1		18.102	3686327	89.34		
2		18.224	439697	10.66		

ODN5 t = 0 h, 25 °C, (detector λ = 440 nm)

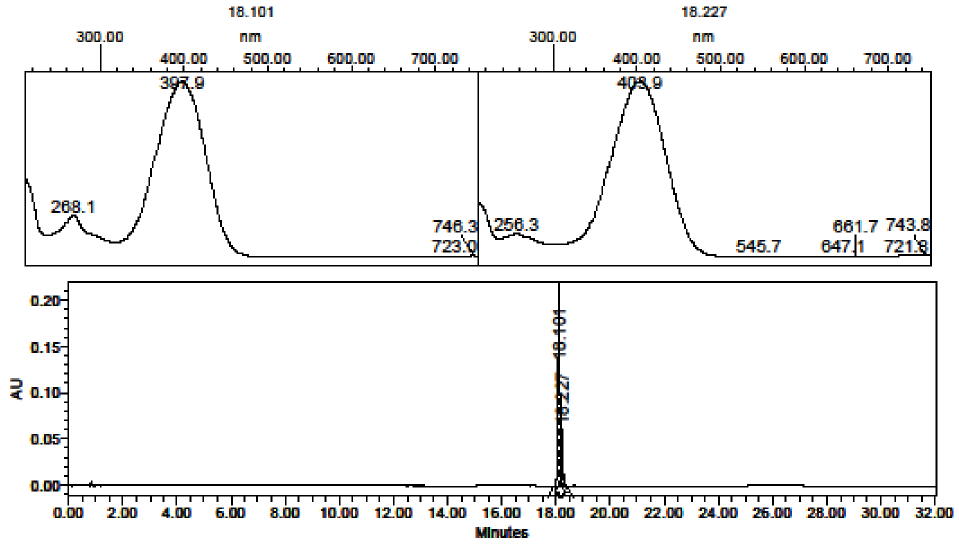

440nm

Pureté et comparaison avec la lirairie

	Name	RT	Area	% Area	Purity1 Angle	Purity1 Threshold
1		18.101	1046533	83.08		
2		18.227	213167	16.92		

ODN5 t = 1 h, 25 °C, λ = (detector λ = 260 nm)

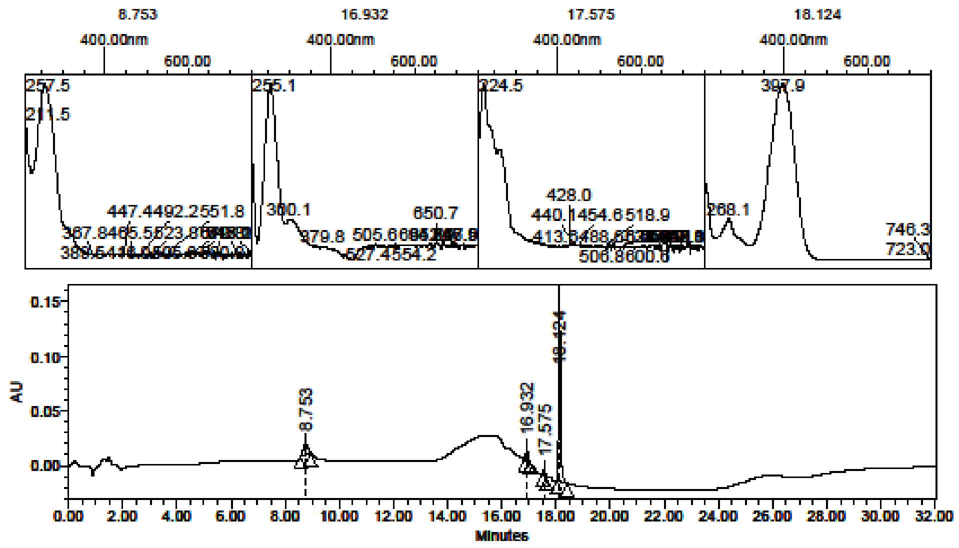

260nm

Pureté et comparaison avec la lirairie

	Name	RT	Area	% Area	Purity1 Angle	Purity1 Threshold
1		8.753	129973	12.51		
2		16.932	45493	4.38		
3		17.575	22974	2.21		
4		18.124	840253	80.90		

ODN5 t = 1 h, 25 °C, (detector λ = 390 nm)

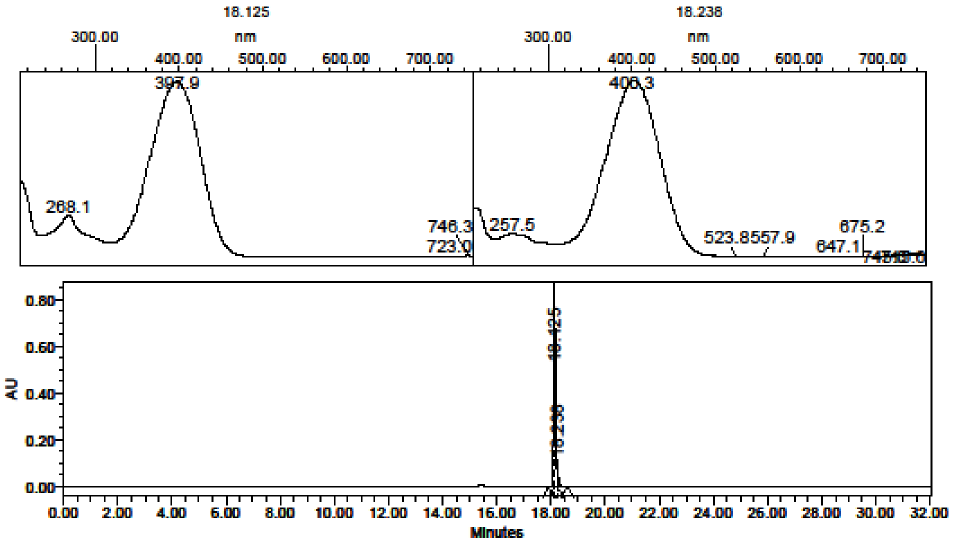

390nm

Pureté et comparaison avec la lirairie

	Name	RT	Area	% Area	Purity1 Angle	Purity1 Threshold
1		18.125	3942829	93.64		
2		18.238	267822	6.36		

ODN5 t = 1 h, 25 °C, (detector λ = 440 nm)

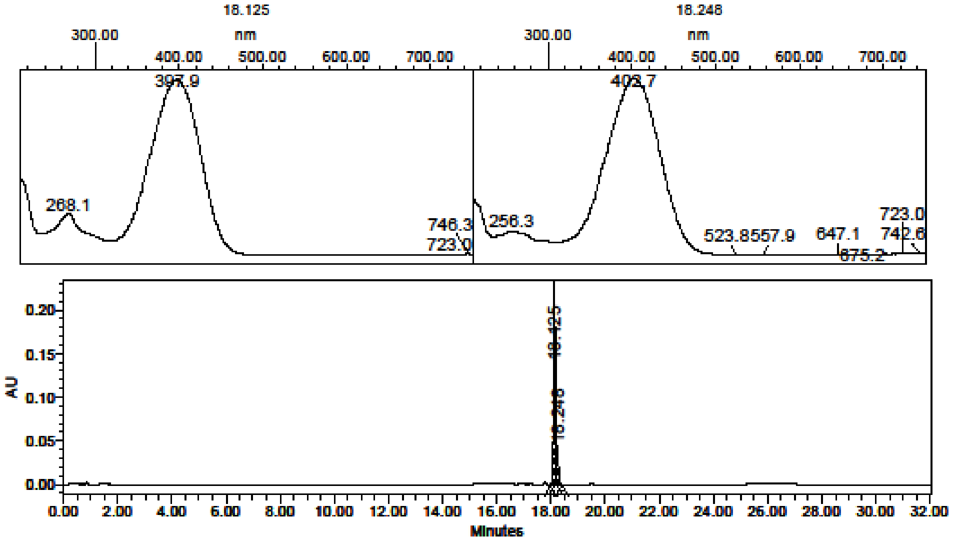

440nm

Pureté et comparaison avec la lirairie

	Name	RT	Area	% Area	Purity1 Angle	Purity1 Threshold
1		18.125	1053289	87.25		
2		18.248	153858	12.75		

ODN5 t = 2 h, 25 °C, (detector λ = 260 nm)

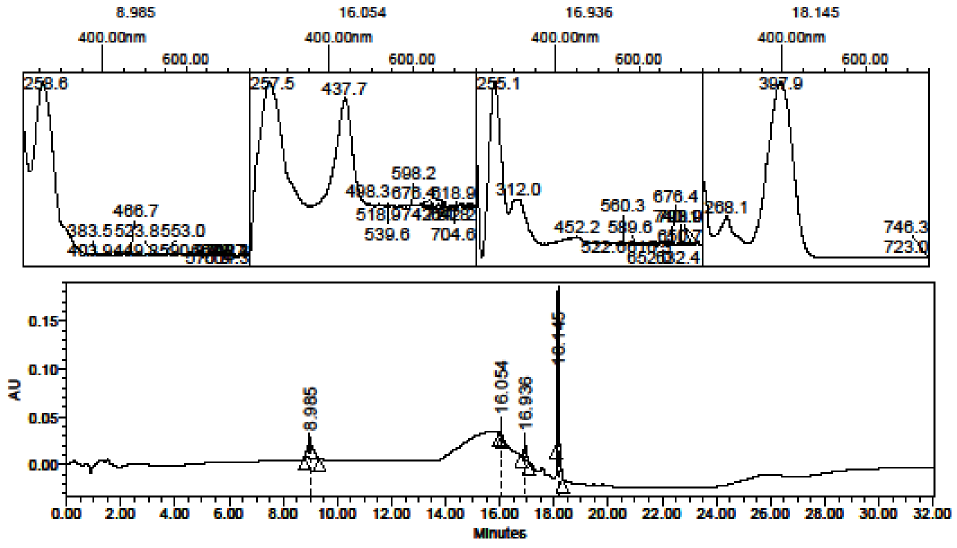

260nm

Pureté et comparaison avec la lirairie

	Name	RT	Area	% Area	Purity1 Angle	Purity1 Threshold
1		8.985	236949	22.21		
2		16.054	25005	2.34		
3		16.936	101731	9.54		
4		18.145	703055	65.91		

ODN5 t = 2 h, 25 °C(detector λ = 390 nm)

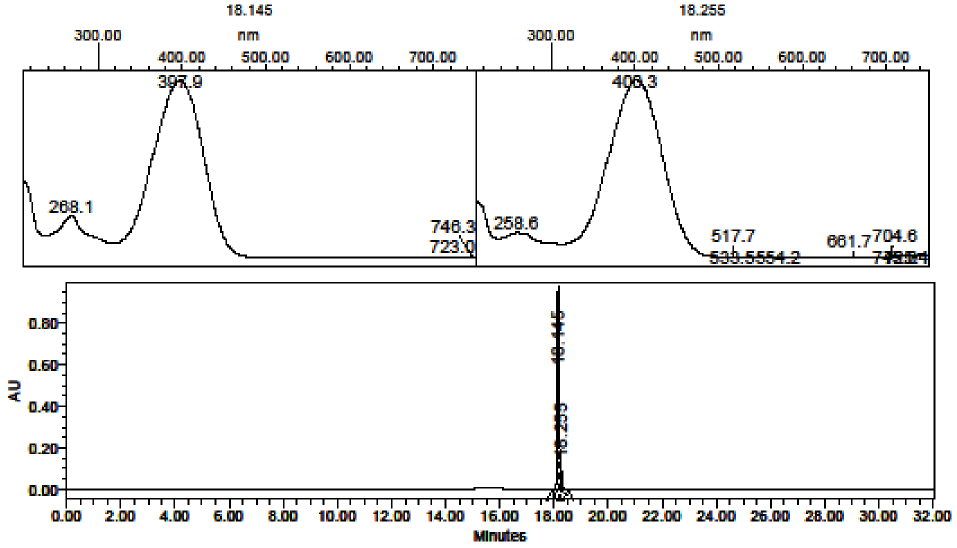

390nm

Pureté et comparaison avec la lirairie

	Name	RT	Area	% Area	Purity1 Angle	Purity1 Threshold
1		18.145	4611742	92.30		
2		18.255	384840	7.70		

ODN5 t = 2 h, 25 °C, (detector λ = 440 nm)

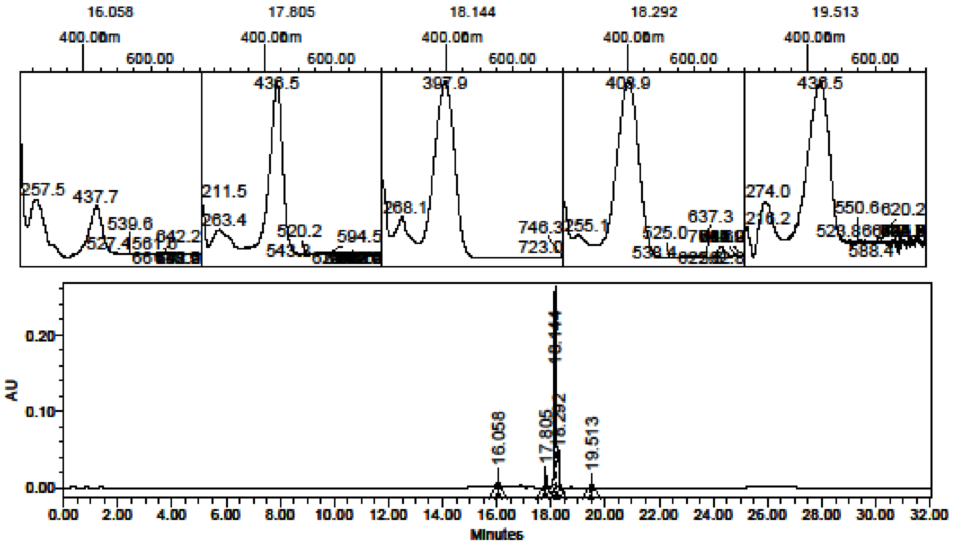

440nm

Pureté et comparaison avec la lirairie

	Name	RT	Area	% Area	Purity1 Angle	Purity1 Threshold
1		16.058	38109	2.51		
2		17.805	56459	3.71		
3		18.144	1345373	88.52		
4		18.292	51892	3.41		
5		19.513	28029	1.84		

ODN5 t = 2 h at 25°C + 1h at 40°C(detector λ = 260 nm)

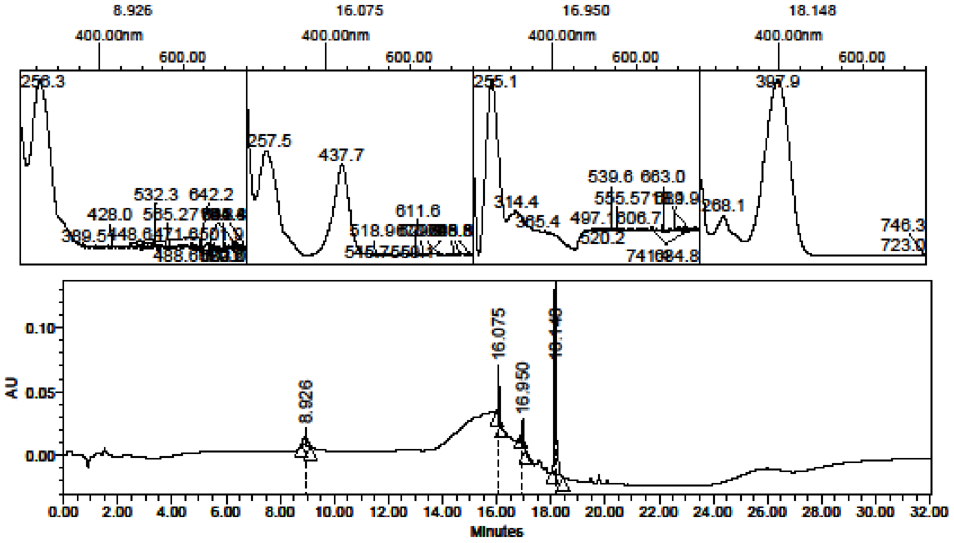

260nm

Pureté et comparaison avec la lirairie

	Name	RT	Area	% Area	Purity1 Angle	Purity1 Threshold
1		8.926	69293	7.20		
2		16.075	131996	13.72		
3		16.950	41447	4.31		
4		18.148	719376	74.77		

ODN5 t = 2 h at 25°C + 1h at 40°C(detector λ = 390 nm)

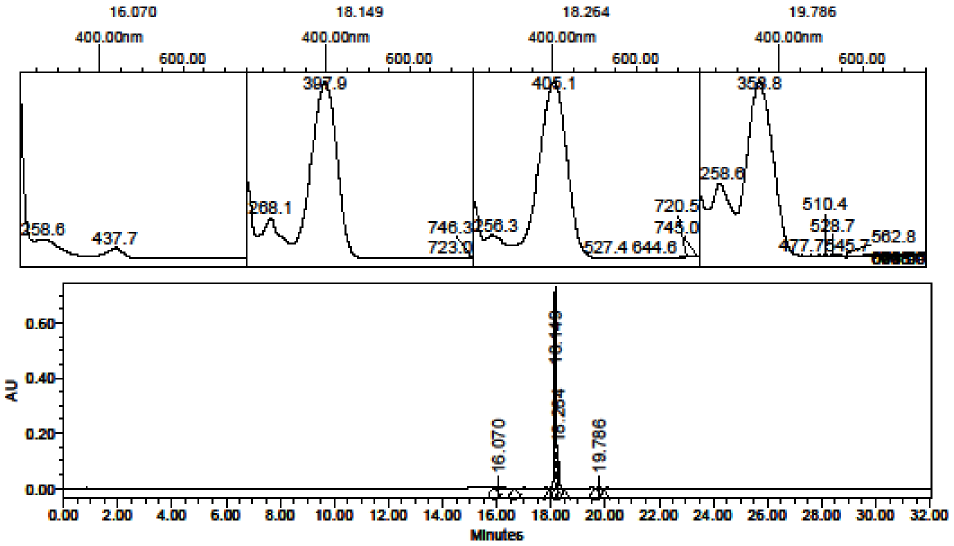

390nm

Pureté et comparaison avec la lirairie

	Name	RT	Area	% Area	Purity1 Angle	Purity1 Threshold
1		16.070	111948	2.75		
2		18.149	3364055	82.66		
3		18.264	550870	13.54		
4		19.786	43030	1.06		

ODN5 t = 2 h at 25°C + 1h at 40°C(detector λ = 440 nm)

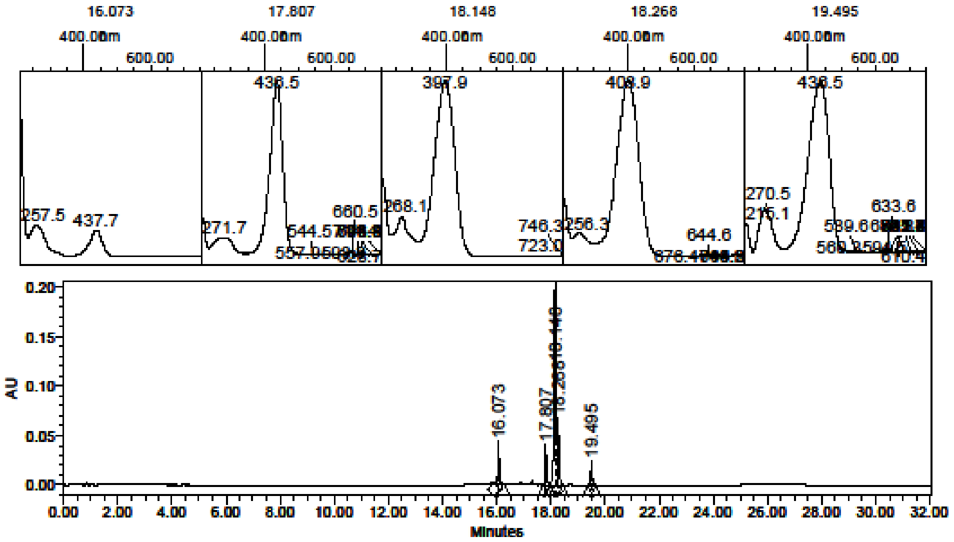

440nm

Pureté et comparaison avec la lirairie

	Name	RT	Area	% Area	Purity1 Angle	Purity1 Threshold
1		16.073	158151	9.63		
2		17.807	134906	8.22		
3		18.148	1008060	61.39		
4		18.268	262999	16.02		
5		19.495	77918	4.75		

ODN5 t = 2 h at 25°C + 2h at 40°C(detector λ = 260 nm)

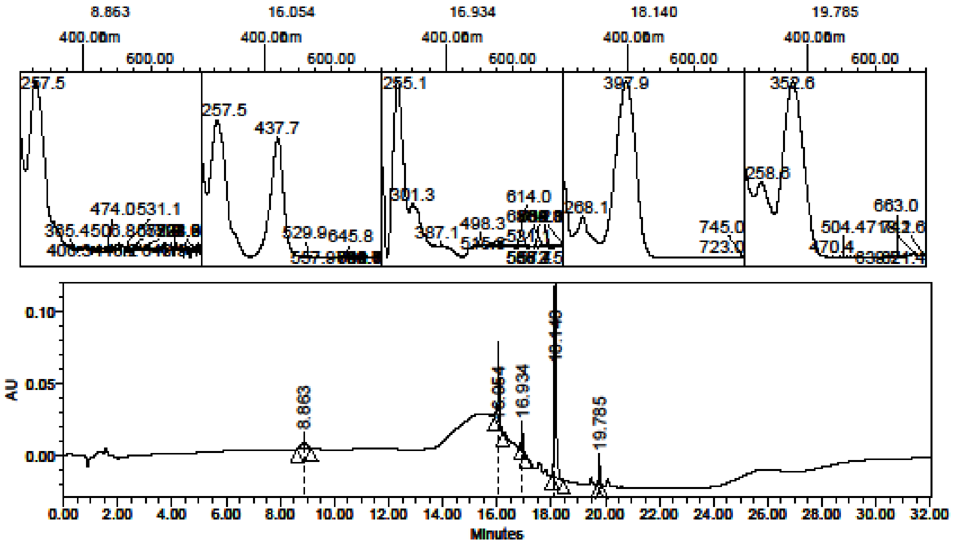

260nm

Pureté et comparaison avec la lirairie

	Name	RT	Area	% Area	Purity1 Angle	Purity1 Threshold
1		8.863	69009	6.45		
2		16.054	231586	21.64		
3		16.934	67531	6.31		
4		18.140	635567	59.39		
5		19.785	66376	6.20		

ODN5 t = 2 h at 25°C + 2h at 40°C (detector λ = 390 nm)

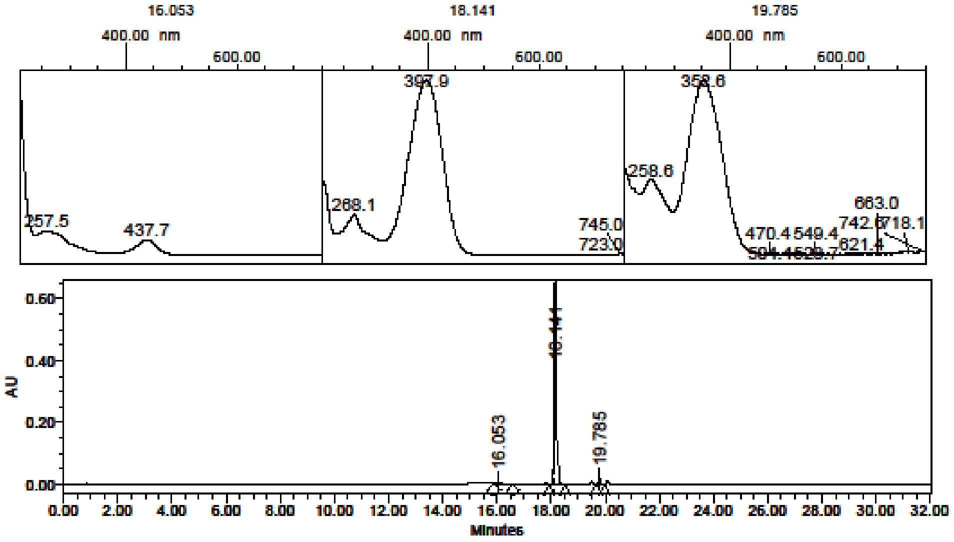

390nm

Pureté et comparaison avec la lirairie

	Name	RT	Area	% Area	Purity1 Angle	Purity1 Threshold
1		16.053	86992	2.55		
2		18.141	3232345	94.88		
3		19.785	87547	2.57		

ODN5 t = 2 h at 25°C + 2h at 40°C (detector λ = 440 nm)

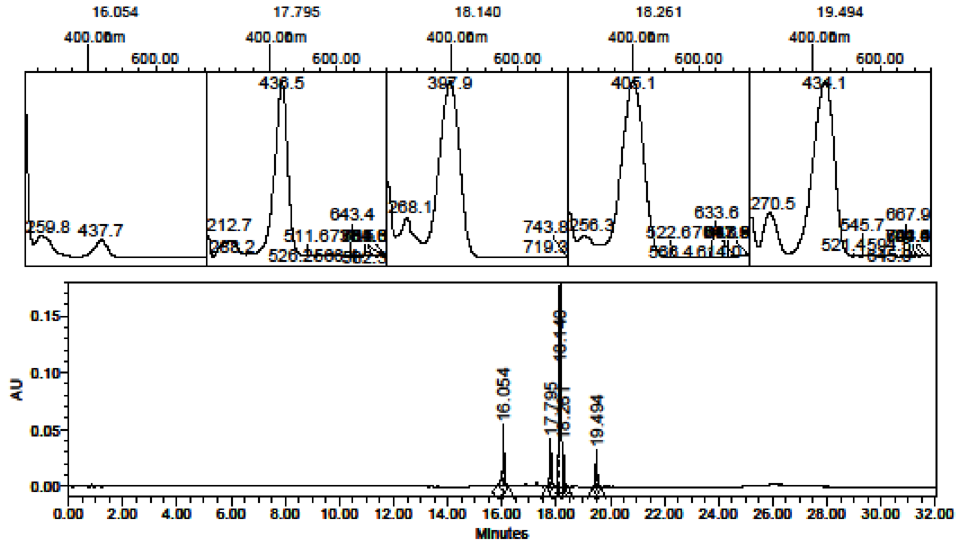

440nm

Pureté et comparaison avec la lirairie

	Name	RT	Area	% Area	Purity1 Angle	Purity1 Threshold
1		16.054	215736	14.53		
2		17.795	146316	9.86		
3		18.140	873630	58.86		
4		18.261	123395	8.31		
5		19.494	125293	8.44		

purified ODN5 (UV detector λ = 260 nm)

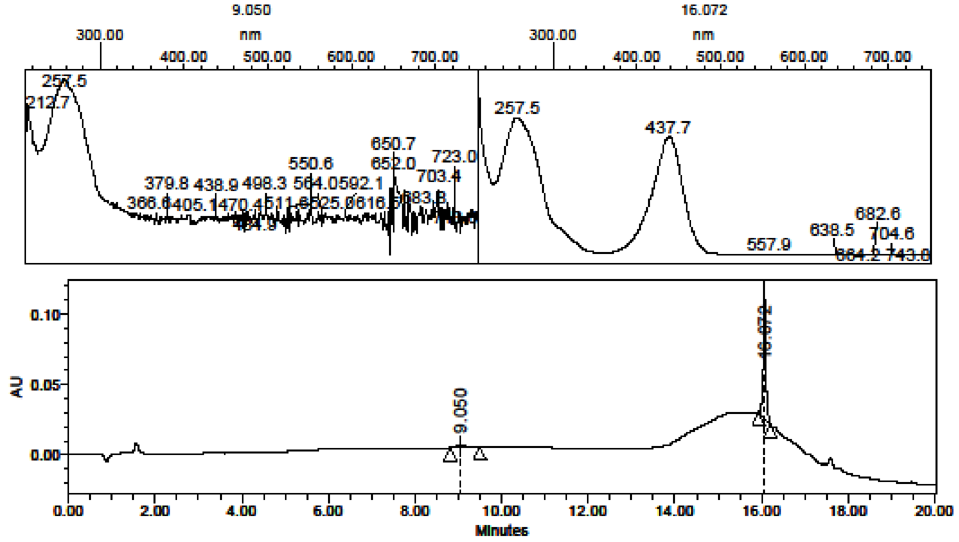

260nm

Pureté et comparaison avec la lirairie

	Name	RT	Area	% Area	Purity1 Angle	Purity1 Threshold
1		9.050	27623	6.53		
2		16.072	395276	93.47		

purified ODN5 (UV detector λ = 390 nm)

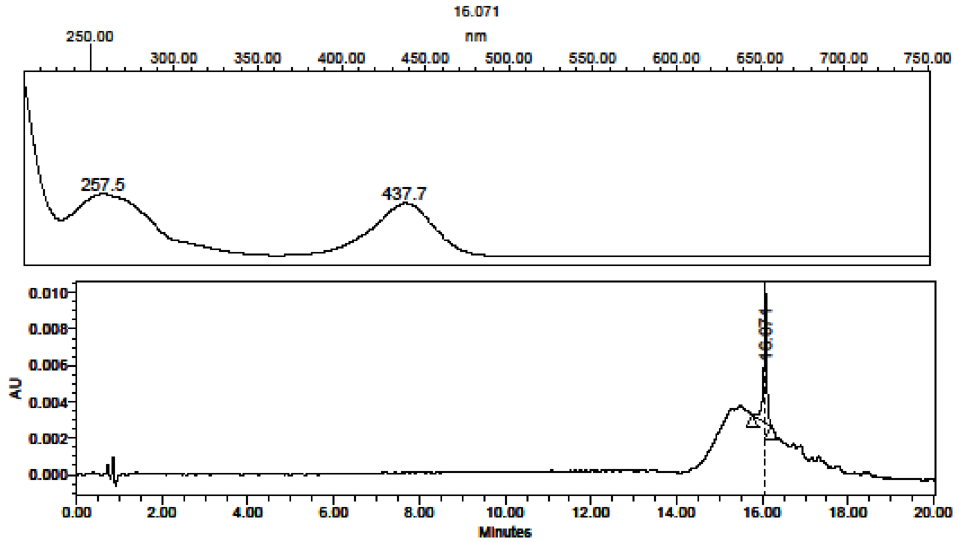

390nm

Pureté et comparaison avec la lirairie

	Name	RT	Area	% Area	Purity1 Angle	Purity1 Threshold
1		16.071	35279	100.00		

purified ODN5 (UV detector λ = 440 nm)

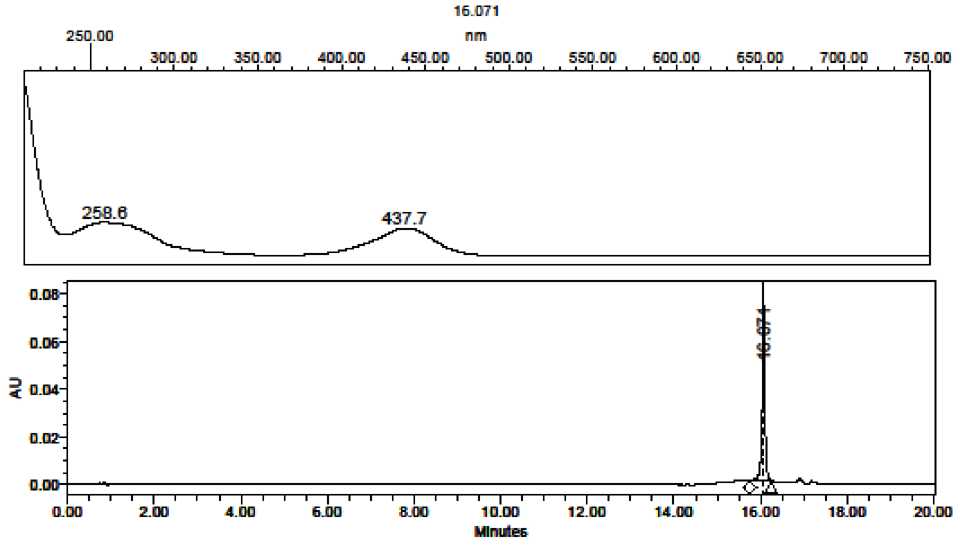

440nm

Pureté et comparaison avec la lirairie

	Name	RT	Area	% Area	Purity1 Angle	Purity1 Threshold
1		16.071	359302	100.00		

S2: UV Spectra of ODN3/5 in Single or Double Strand

Conjugates UV-Vis Properties

[ODN] = 5.96 10^{-6} M in phosphate buffer (10 mM Na_2HPO_4, 100 mM NaCl, 1 mM EDTA, pH = 7)

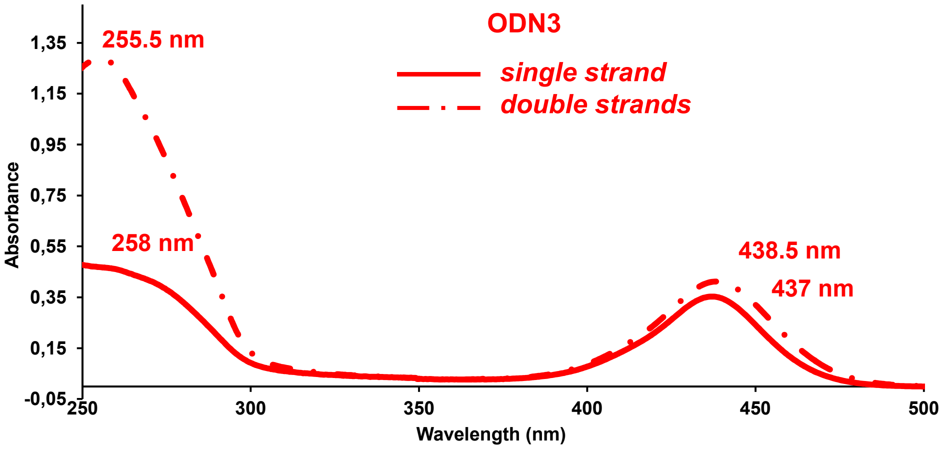

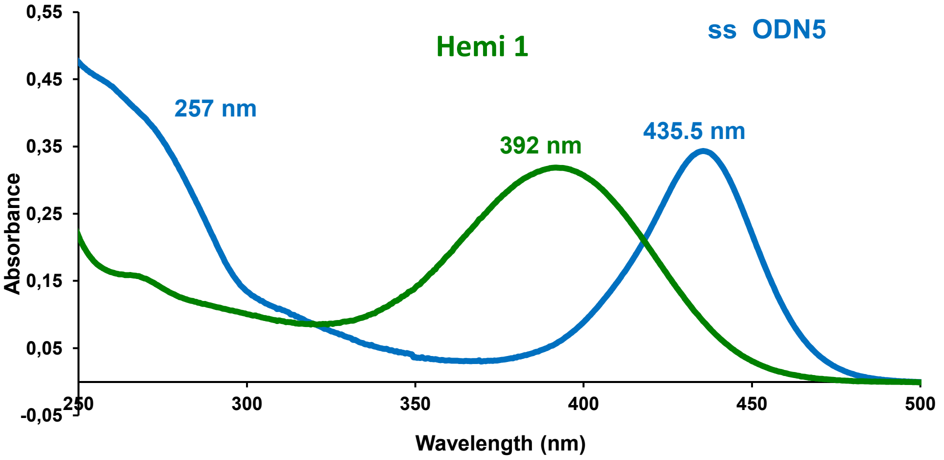

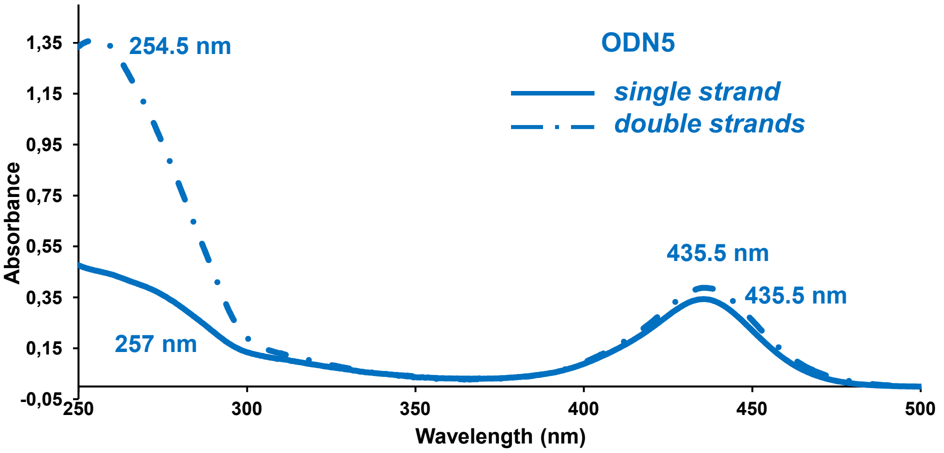

Conjugates Fluorescence Properties

Emission fluorescence spectra λ_{ex} = 420 nm
[ODN] = 1.49 10^{-6} M in phosphate buffer (10 mM Na_2HPO_4, 100 mM NaCl, 1 mM EDTA, pH = 7)

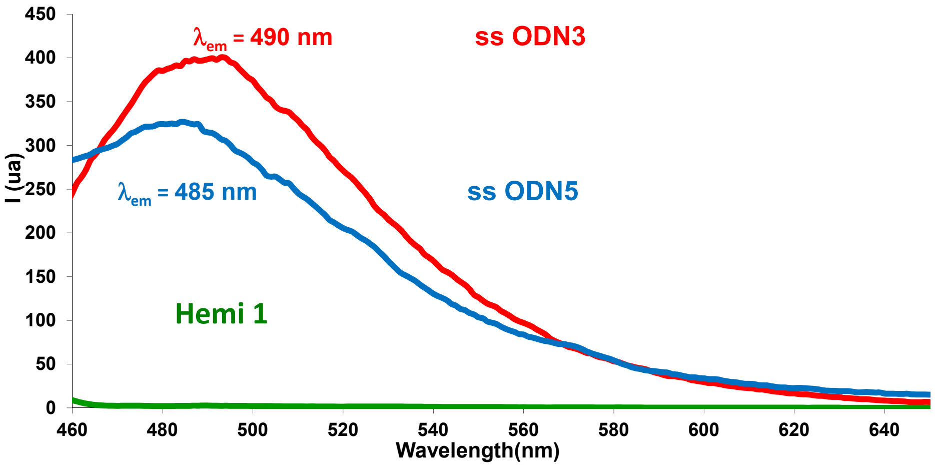

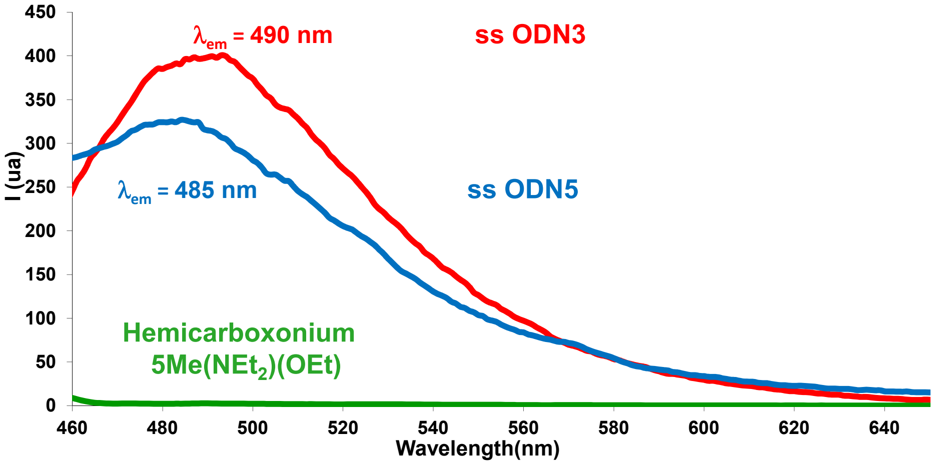

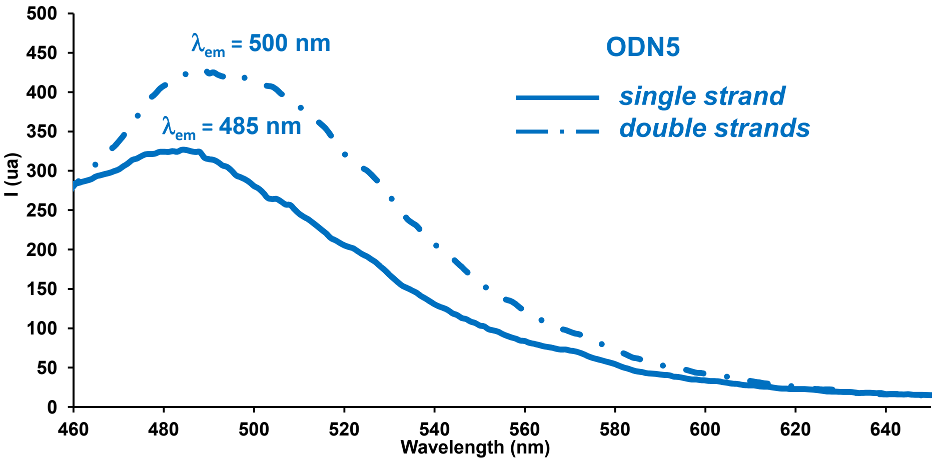

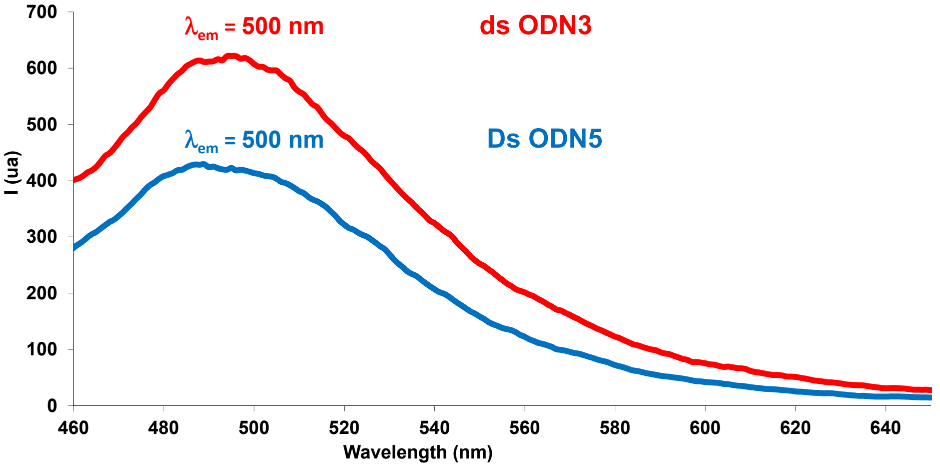

S3: Mass spectra of ODN2, ODN4, ODN3 and ODN5

ODN2

These analyses were recorded in a row with THAP matrix on a MALDI-Tof spectrophotometer WATERS Micro-MX.

	Exact mass [M]	Expected mass m/z = [M-H]$^{+}$	Obtained mass	% error
**T10**	2978.50	2977.50	2973.3	0.12%
**T14**	4194.69	4193.69	4192.2	0.035%
**ODN2**	3101.61	3100.61	3097.9	0.06%

T10

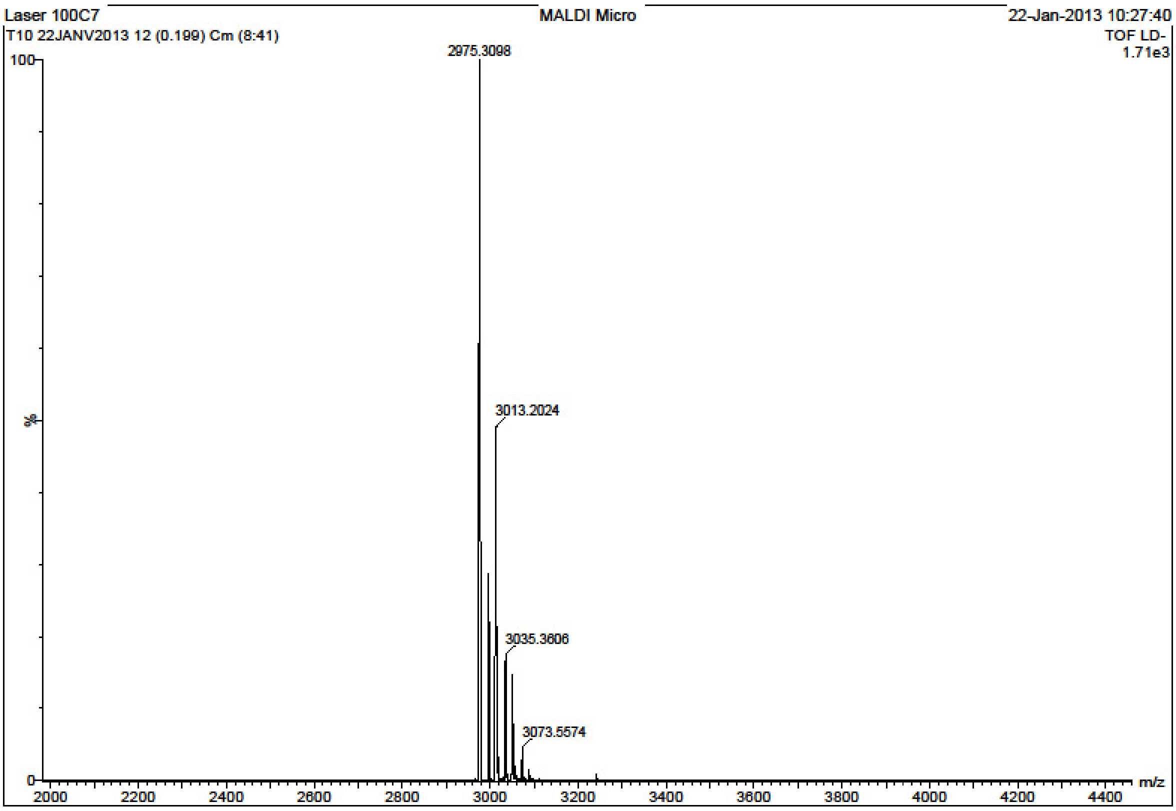

T14

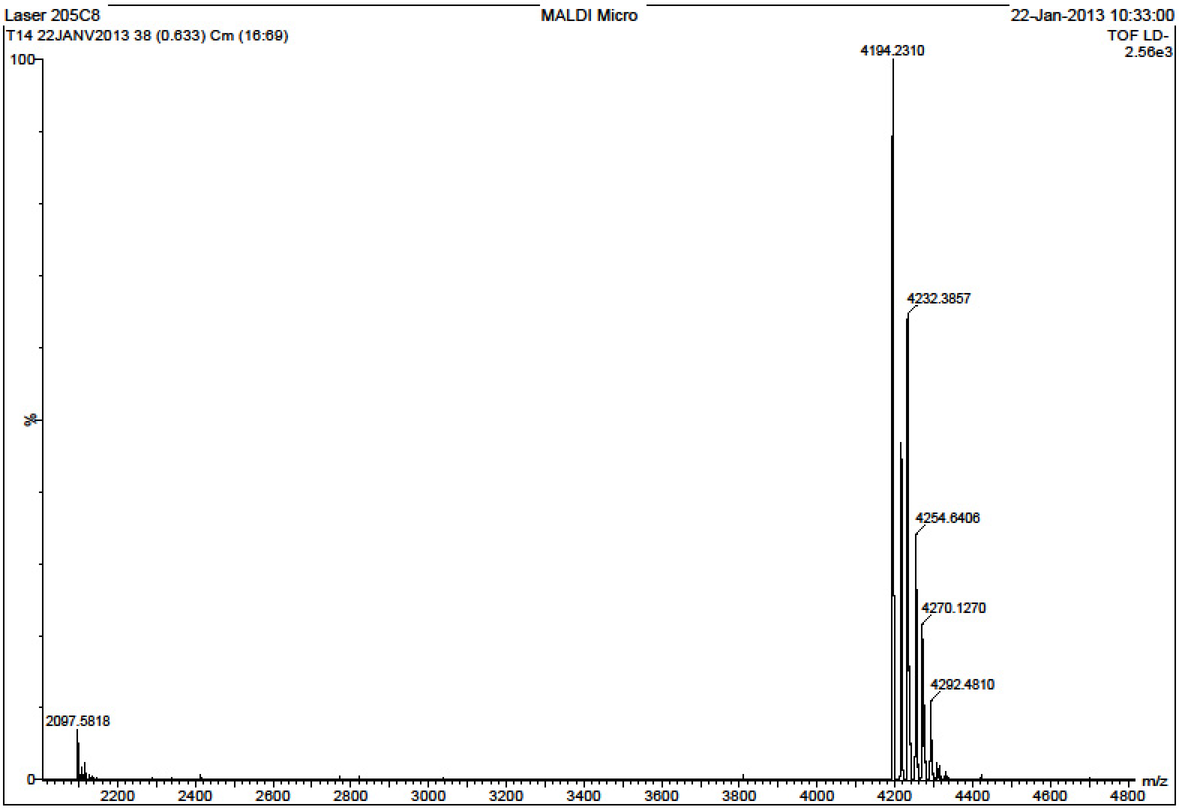
Laser 205C8
MALDI Micro
22-Jan-2013 10:33:00
T14 22JANV2013 38 (0.633) Cm (16:69)
TOF LD-
2.56e3
100
%
0
4194.2310
4232.3857
4254.6406
4270.1270
4292.4810
2097.5818
2200
2400
2600
2800
3000
3200
3400
3600
3800
4000
4200
4400
4600
4800
m/z

ODN2

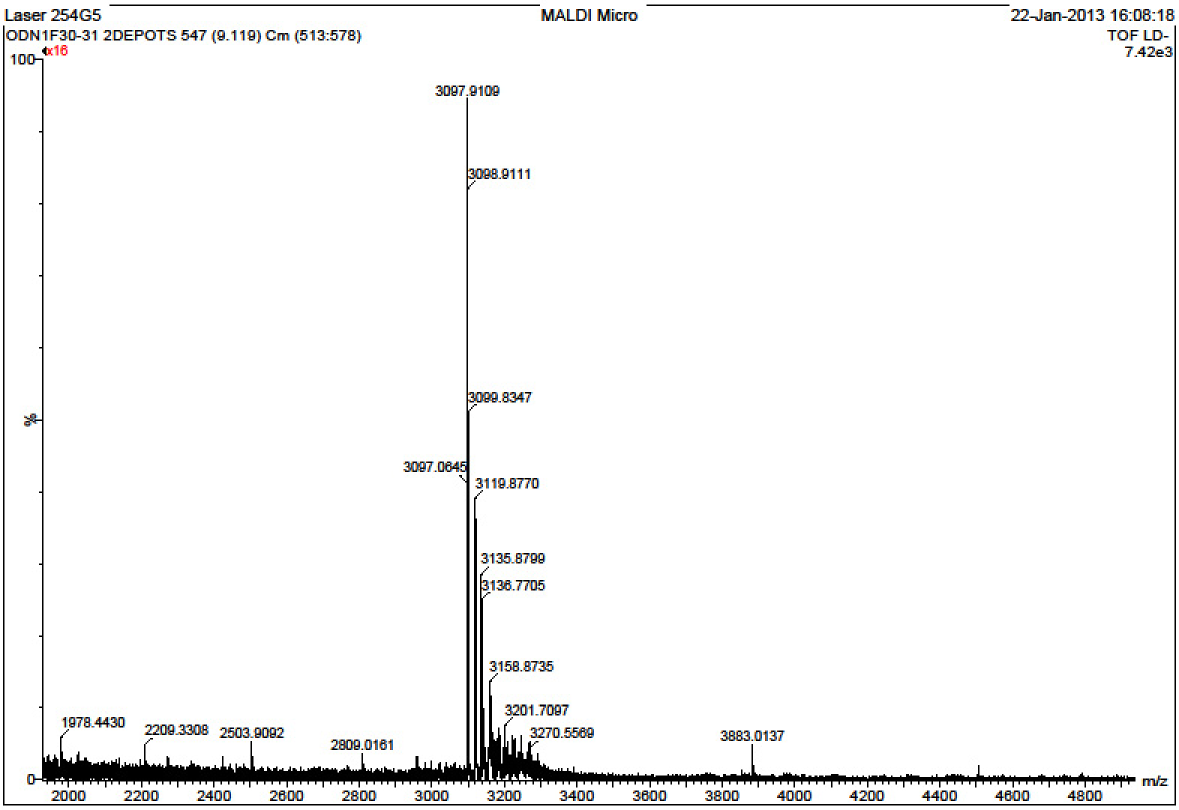
Laser 254G5
MALDI Micro
22-Jan-2013 16:08:18
ODN1F30-31 2DEPOTS 547 (9.119) Cm (513:578)
TOF LD-
7.42e3
100
x16
%
0
3097.9109
3098.9111
3099.8347
3097.0645
3119.8770
3135.8799
3136.7705
3158.8735
3201.7097
3270.5569
3883.0137
1978.4430
2209.3308
2503.9092
2809.0161
2000
2200
2400
2600
2800
3000
3200
3400
3600
3800
4000
4200
4400
4600
4800
m/z

ODN4

These analyses were recorded in a row with THAP matrix on a MALDI-Tof spectrophotometer WATERS Micro-MX

	Exact mass [M]	Expected mass m/z = [M-H]$^+$	Obtained mass	% error
**T10**	2978.50	2977.50	2969.6	0.26 %
**T14**	4194.69	4193.69	4179.0	0.35 %
**ODN4**	3172.64	3171.64	3161.7	0.31 %

T10

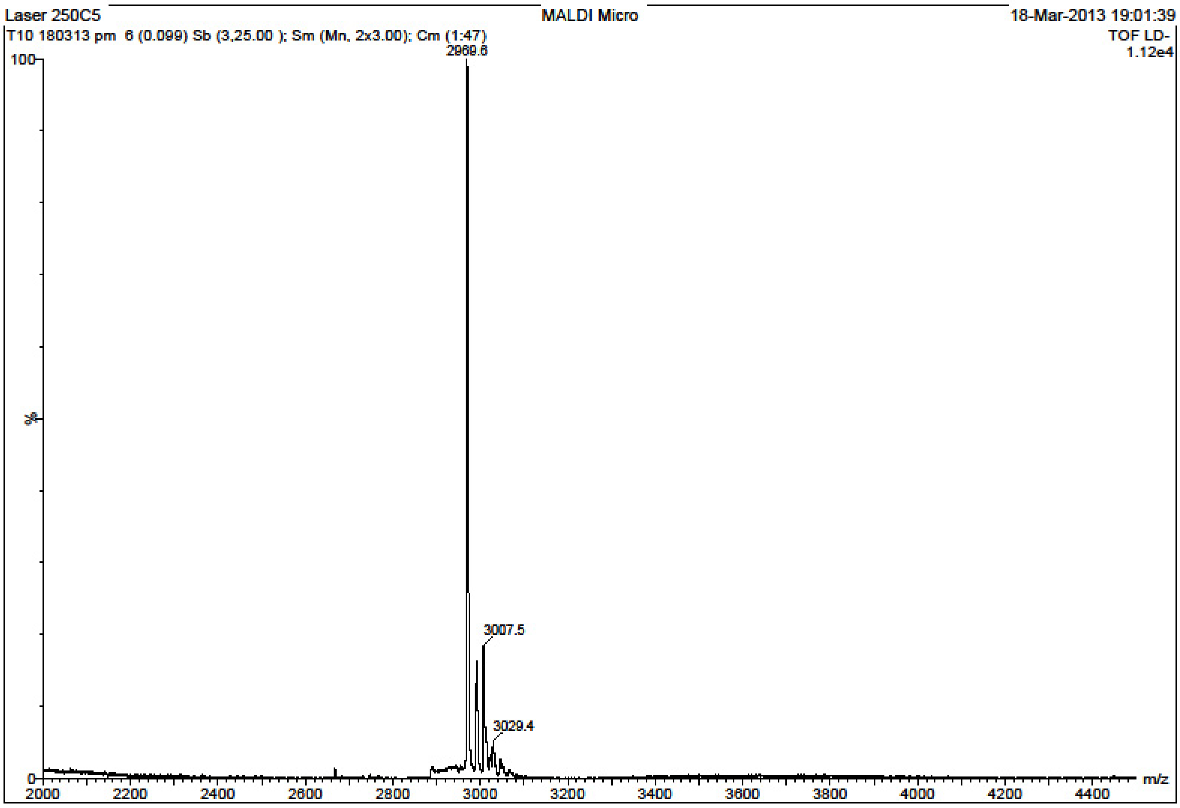

T14

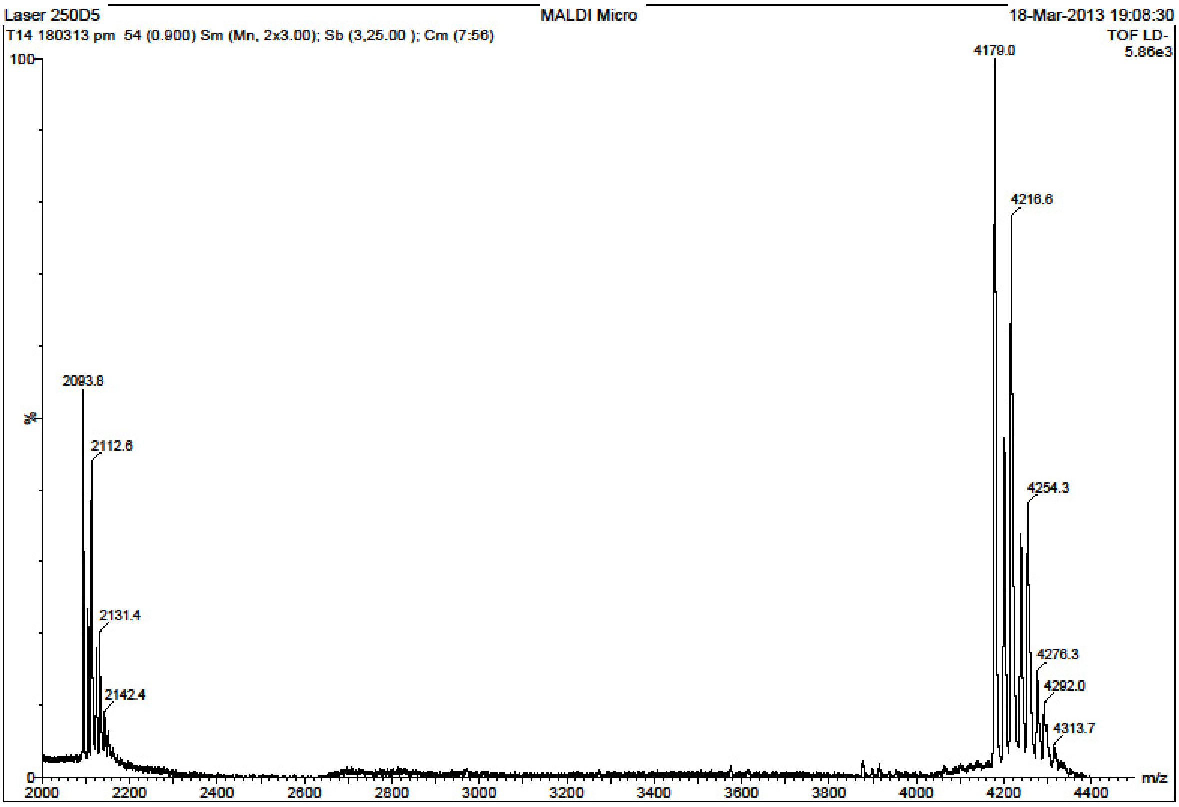
Laser 250D5
MALDI Micro
18-Mar-2013 19:08:30
T14 180313 pm 54 (0.900) Sm (Mn, 2x3.00); Sb (3,25.00); Cm (7:56)
TOF LD-
5.86e3
4179.0
4216.6
4254.3
4276.3
4292.0
4313.7
2093.8
2112.6
2131.4
2142.4
%
m/z

ODN4

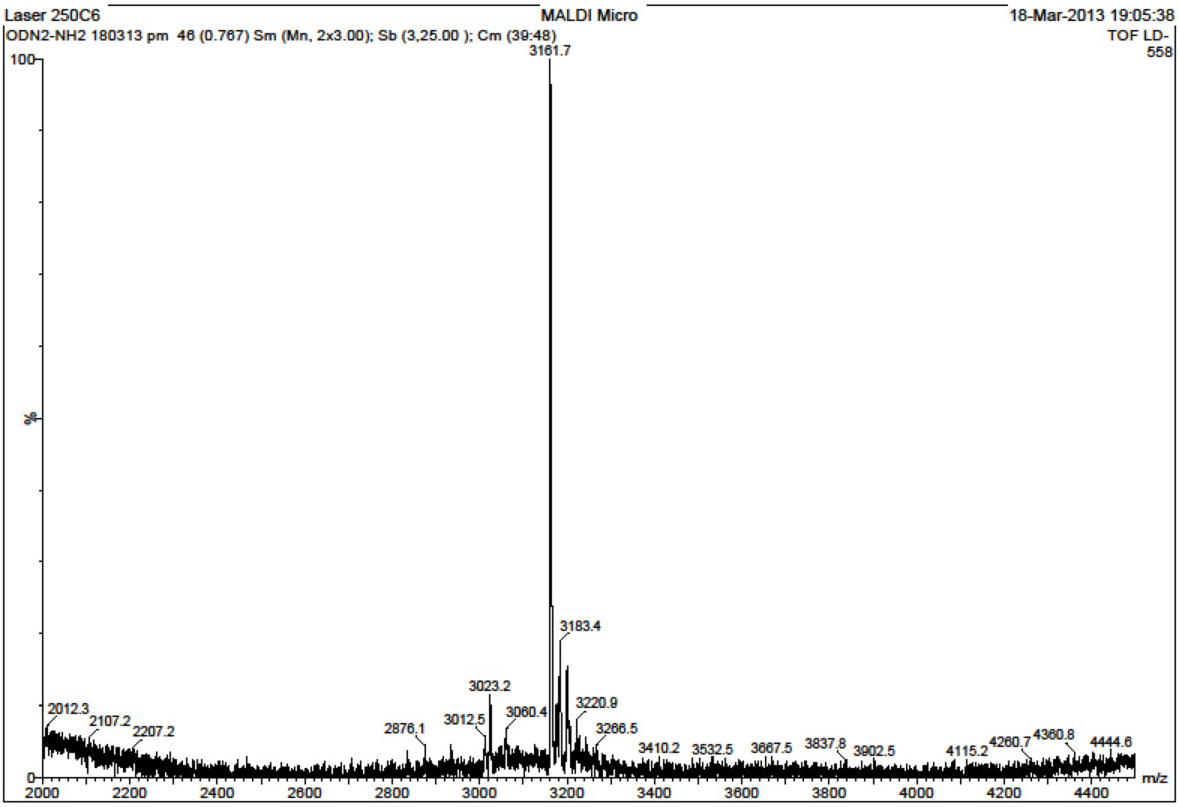
Laser 250C6
MALDI Micro
18-Mar-2013 19:05:38
ODN2-NH2 180313 pm 46 (0.767) Sm (Mn, 2x3.00); Sb (3,25.00); Cm (39:48)
TOF LD-
558
3161.7
3183.4
3023.2
3220.9
3060.4
3012.5
3266.5
2876.1
2012.3
2107.2
2207.2
3410.2
3532.5
3667.5
3837.8
3902.5
4115.2
4260.7
4360.8
4444.6
%
m/z

ODN3 and ODN5

These analyses were recorded in a row with THAP matrix on a MALDI-Tof spectrophotometer WATERS Micro-MX.

	Exact mass [M]	Expected mass m/z	Obtained mass	% error
**T10**	2978.50	$[M-H]^+$ 2977.50	2969.3	0.27%
**T14**	4194.69	$[M-H]^+$ 4193.69	4176.2	0.41%
**ODN3**	3417.81	$[M-2H]^+$ 3415.81	3403.8	0.35%
**ODN5**	3488.85	$[M-2H]^+$ 3486.85	3475.3	0.33%

T10

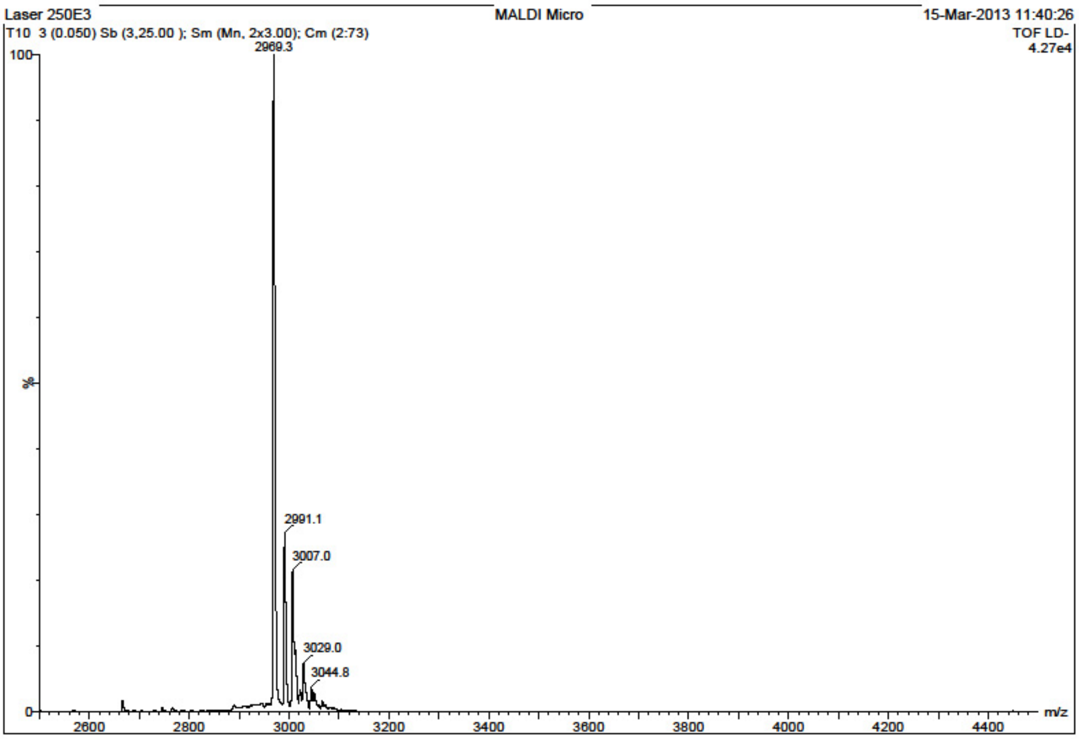

T14

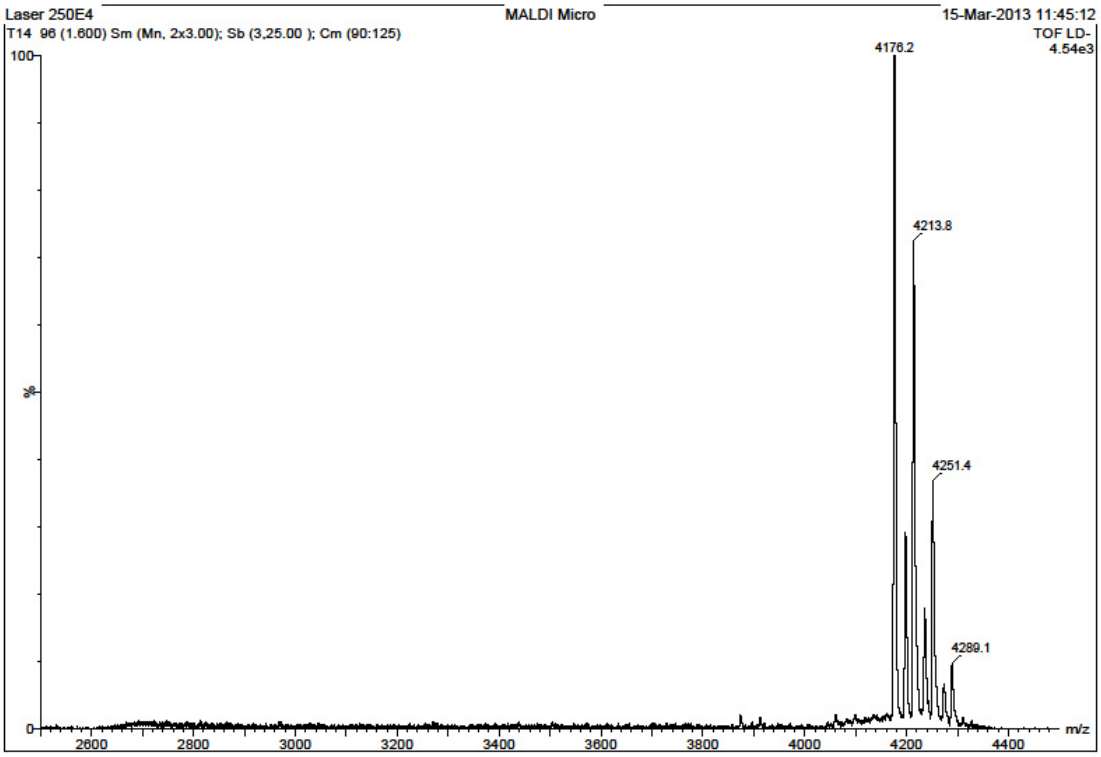
Laser 250E4
MALDI Micro
15-Mar-2013 11:45:12
T14 96 (1.600) Sm (Mn, 2x3.00); Sb (3,25.00); Cm (90:125)
TOF LD-
4.54e3
4176.2
4213.8
4251.4
4289.1
100
%
0
2600
2800
3000
3200
3400
3600
3800
4000
4200
4400
m/z

ODN3

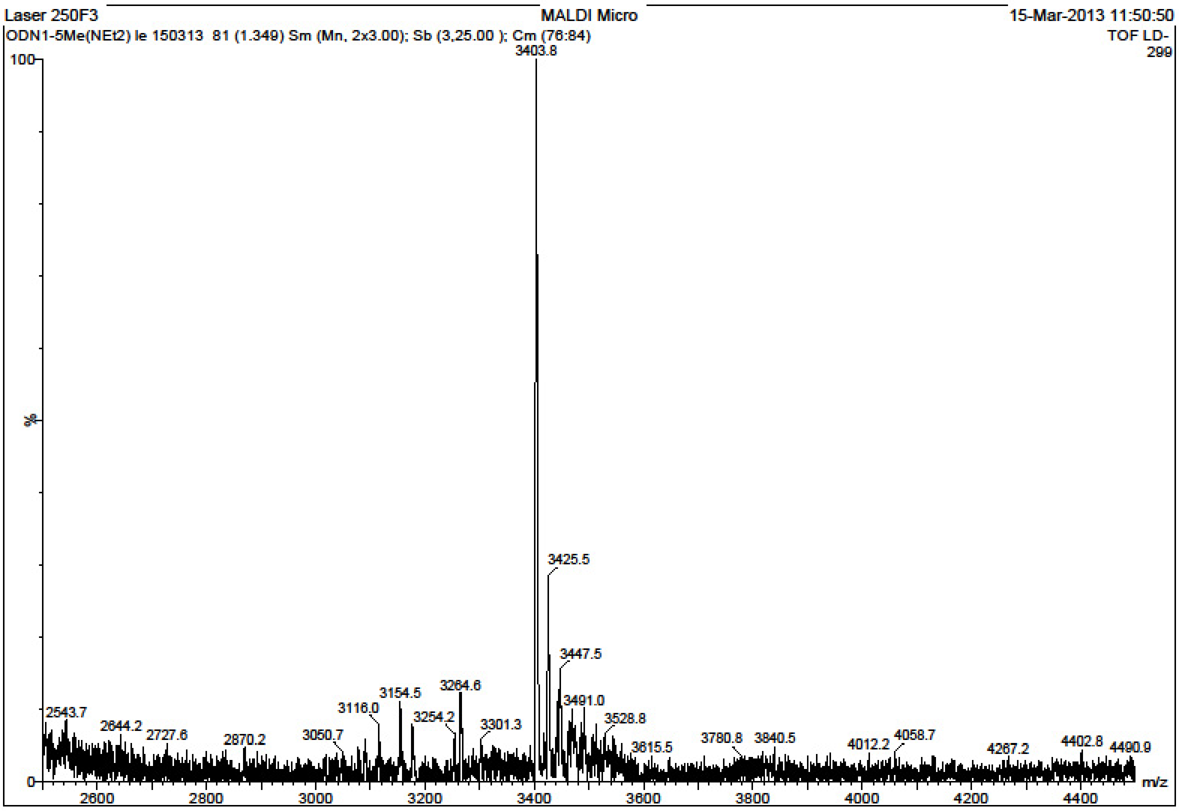
Laser 250F3
MALDI Micro
15-Mar-2013 11:50:50
ODN1-5Me(NEt2) le 150313 81 (1.349) Sm (Mn, 2x3.00); Sb (3,25.00); Cm (76:84)
TOF LD-
299
3403.8
3425.5
3447.5
3491.0
3528.8
3615.5
3780.8
3840.5
4012.2
4058.7
4267.2
4402.8
4490.9
2543.7
2644.2
2727.6
2870.2
3050.7
3116.0
3154.5
3254.2
3264.6
3301.3
100
%
0
2600
2800
3000
3200
3400
3600
3800
4000
4200
4400
m/z

ODN5

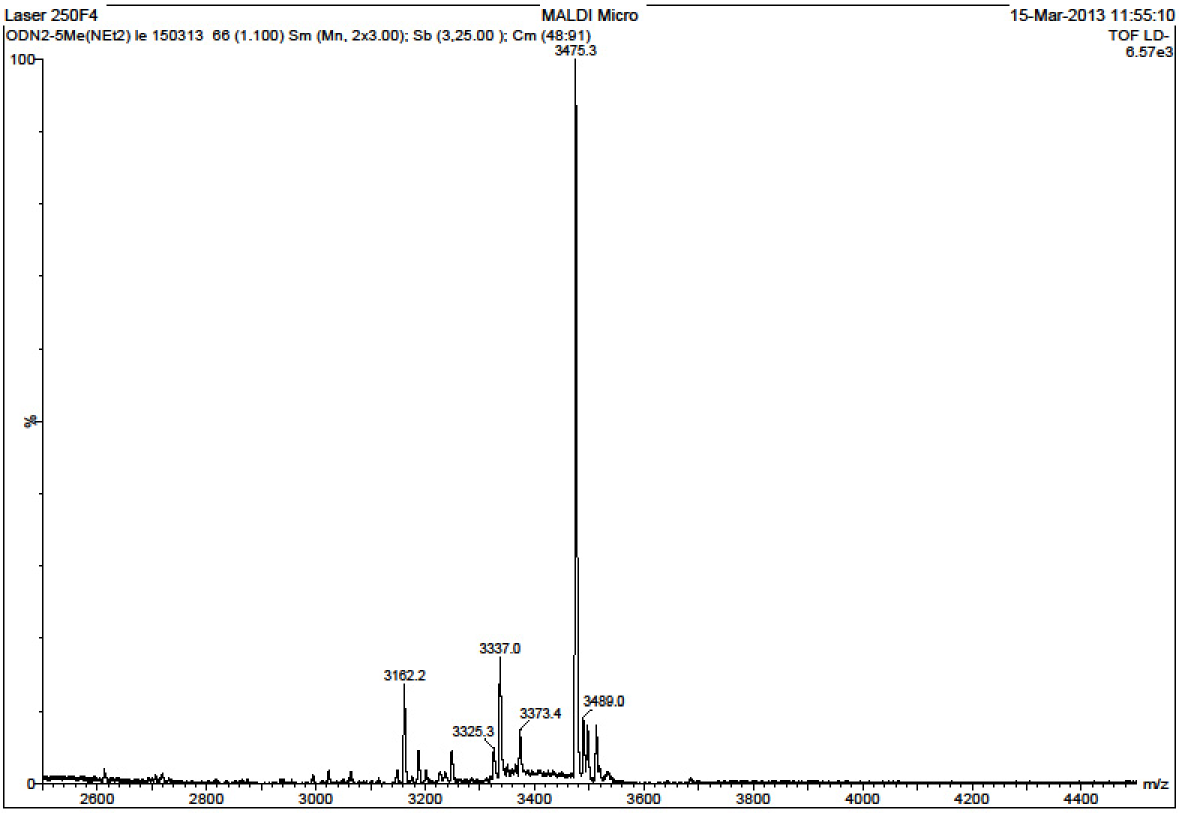
Laser 250F4
MALDI Micro
15-Mar-2013 11:55:10
ODN2-5Me(NEt2) le 150313 66 (1.100) Sm (Mn, 2x3.00); Sb (3,25.00); Cm (48:91)
TOF LD-
6.57e3
3475.3
3337.0
3162.2
3489.0
3373.4
3325.3
100
%
0
2600
2800
3000
3200
3400
3600
3800
4000
4200
4400
m/z

S4: Melting Temperature of ODN3 and ODN5 within Duplex

The thermal denaturation curves were recorded on a Varian CARY-300 Bio UV-vis spectrophotometer with the Cary Win UV thermal software. All measurements were performed in 1.0 cm path-length micro cuvettes in 10 mM Na_2HPO_4, 100 mM NaCl, 1 mM EDTA, pH = 7, buffer. The concentration of the ODNs were calculated by measuring the absorbance at 260nm and at 80°C. Sample were heated at 90°C and cooled to 10°C at a rate of 0.5 °C/min and then warm up to 90°C at the same rate.

	**Sequences**	**T_m °C (ΔT_m)**
**unmodified ADN/ADNc**	$_{5'}$-d(GCG CTT GCC G)-$_{3'}$/$_{5'}$-d(CGG CAA GCG C)-$_{3'}$	**53.0**
**unmodified ADN/ARNc**	$_{5'}$-d(GCG CTT GCC G)-$_{3'}$/$_{5'}$-r(CGG CAA GCG C)-$_{3'}$	**55.2**
**ODN3/ADNc**	$_{5'}$-d(GCG CTT GCC G)-$_{3'}$/$_{5'}$-d(CGG CAA GCG C)-$_{3'}$	**52.0 (-1.0)**
**ODN3/RcT4**	$_{5'}$-d(GCG CTT GCC G)-$_{3'}$/$_{5'}$-d(CGG TAA GCG C)-$_{3'}$	**39.0 (-14.0)**
**ODN3/RcT5**	$_{5'}$-d(GCG CTT GCC G)-$_{3'}$/$_{5'}$-d(CGG CTA GCG C)-$_{3'}$	**43.8 (-10.0)**
**ODN3/RcT6**	$_{5'}$-d(GCG CTT GCC G)-$_{3'}$/$_{5'}$-d(CGG CAT GCG C)-$_{3'}$	**46.0 (-7.0)**
**ODN3/RcT7**	$_{5'}$-d(GCG CTT GCC G)-$_{3'}$/$_{5'}$-d(CGG CAA TCG C)-$_{3'}$	**Nd (nd)**
**ODN3/ARNc**	$_{5'}$-d(GCG CTT GCC G)-$_{3'}$/$_{5'}$-r(CGC CAA GCG C)-$_{3'}$	**50 .0 (-5.2)**
**ODN3/RcU4**	$_{5'}$-d(GCG CTT GCC G)-$_{3'}$/$_{5'}$-r(CGG UAA GCG C)-$_{3'}$	**34.0 (-21.2)**
**ODN3/RcU5**	$_{5'}$-d(GCG CTT GCC G)-$_{3'}$/$_{5'}$-r(CGG CUA GCG C)-$_{3'}$	**50.0 (-5.2)**
**ODN3/RcU6**	$_{5'}$-d(GCG CTT GCC G)-$_{3'}$/$_{5'}$-r(CGG CAU GCG C)-$_{3'}$	**49.0 (-6.2)**
**ODN3/RcU7**	$_{5'}$-d(GCG CTT GCC G)-$_{3'}$/$_{5'}$- r(CGG CAA UCG C)-$_{3'}$	**48.0 (-7.2)**
**ODN5/ADNc**	$_{5'}$-d(GCG CTT GCC G)-$_{3'}$/$_{5'}$-d(CGG CAA GCG C)-$_{3'}$	**52.0 (-1.0)**
**ODN5/RcT4**	$_{5'}$-d(GCG CTT GCC G)-$_{3'}$/$_{5'}$-d(CGG TAA GCG C)-$_{3'}$	**38.0 (-15.0)**
**ODN5/RcT5**	$_{5'}$-d(GCG CTT GCC G)-$_{3'}$/$_{5'}$-d(CGG CTA GCG C)-$_{3'}$	**45.0 (-8.0)**
**ODN5/RcT6**	$_{5'}$-d(GCG CTT GCC G)-$_{3'}$/$_{5'}$-d(CGG CAT GCG C)-$_{3'}$	**45.0 (-8.0)**
**ODN5/RcT7**	$_{5'}$-d(GCG CTT GCC G)-$_{3'}$/$_{5'}$-d(CGG CAA TCG C)-$_{3'}$	**Nd (nd)**
**ODN5/ARNc**	$_{5'}$-d(GCG CTT GCC G)-$_{3'}$/$_{5'}$- r(CGC CAA GCG C)-$_{3'}$	**56.0 (+0.8)**
**ODN5/RcU4**	$_{5'}$-d(GCG CTT GCC G)-$_{3'}$/$_{5'}$- r(CGG UAA GCG C)-$_{3'}$	**40.0 (-15.2)**
**ODN5/RcU5**	$_{5'}$-d(GCG CTT GCC G)-$_{3'}$/$_{5'}$- r(CGG CUA GCG C)-$_{3'}$	**50.0 (-5.2)**
**ODN5/RcU6**	$_{5'}$-d(GCG CTT GCC G)-$_{3'}$/$_{5'}$- r(CGG CAU GCG C)-$_{3'}$	**48.0 (-7.2)**
**ODN5/RcU7**	$_{5'}$-d(GCG CTT GCC G)-$_{3'}$/$_{5'}$- r(CGG CAA UCG C)-$_{3'}$	**50.0 (-5.2)**

Nd: no transition detected
